# Systematic identification of potential key microRNAs and circRNAs in the dorsal root ganglia of mice with sciatic nerve injury

**DOI:** 10.3389/fnmol.2023.1119164

**Published:** 2023-03-14

**Authors:** Youfen Yu, Xueru Xu, Chun Lin, Rongguo Liu

**Affiliations:** ^1^Department of Pain Management, Fujian Provincial Hospital, Shengli Clinical Medical College of Fujian Medical University, Fuzhou, China; ^2^Institute of Pain Research, School of Basic Medical Sciences, Fujian Medical University, Fuzhou, Fujian, China

**Keywords:** neuropathic pain, microRNA, circRNA, ceRNA, dorsal root ganglia, miR-181a-5p, miR-223-3p

## Abstract

**Background:**

Neuropathic pain (NeP) is a pathological condition arising from a lesion or disease affecting the somatosensory system. Accumulating evidence has shown that circular RNAs (circRNAs) exert critical functions in neurodegenerative diseases by sponging microRNAs (miRNAs). However, the functions and regulatory mechanisms of circRNAs as competitive endogenous RNAs (ceRNAs) in NeP remain to be determined.

**Methods:**

The sequencing dataset GSE96051 was obtained from the public Gene Expression Omnibus (GEO) database. First, we conducted a comparison of gene expression profiles in the L3/L4 dorsal root ganglion (DRG) of sciatic nerve transection (SNT) mice (*n* = 5) and uninjured mice (Control) (*n* = 4) to define the differentially expressed genes (DEGs). Then, critical hub genes were screened by exploring protein–protein interaction (PPI) networks with Cytoscape software, and the miRNAs bound to them were predicted and selected and then validated by qRT-PCR. Furthermore, key circRNAs were predicted and filtered, and the network of circRNA-miRNA-mRNA in NeP was constructed.

**Results:**

A total of 421 DEGs were identified, including 332 upregulated genes and 89 downregulated genes. Ten hub genes, including IL6, Jun, Cd44, Timp1, and Csf1, were identified. Two miRNAs, mmu-miR-181a-5p and mmu-miR-223-3p, were preliminarily verified as key regulators of NeP development. In addition, circARHGAP5 and circLPHN3 were identified as key circRNAs. Gene Ontology (GO) and Kyoto Encyclopedia of Genes and Genomes (KEGG) analysis demonstrated that these differentially expressed mRNAs and targeting miRNAs were involved in signal transduction, positive regulation of receptor-mediated endocytosis and regulation of neuronal synaptic plasticity. These findings have useful implications for the exploration of new mechanisms and therapeutic targets for NeP.

**Conclusion:**

These newly identified miRNAs and circRNAs in networks reveal potential diagnostic or therapeutic targets for NeP.

## 1. Introduction

Neuropathic pain (NeP) is defined as chronic pain that arises from a lesion or disease that affects the somatosensory system by the International Association for the Study of Pain (IASP). NeP manifests as hyperalgesia, allodynia, and spontaneous pain, which have serious impacts on people’s daily lives. The most common clinical NeP diseases include trigeminal neuralgia, postherpetic neuralgia, and painful radiculopathy (Scholz et al., 2019). The common critical mechanism of different NeP diseases involves peripheral and central sensitization. Specific pathophysiological changes are involved in the pathogenesis of NeP, such as alterations in ion channel activity, activation of microglia and epigenetic modulation of nerve cells (Penas and Navarro, 2018).

It has been well accepted that the dorsal root ganglion (DRG) plays an important role in nociceptive transmission and modulation. In particular, the DRG receives peripheral nociceptive information and transmits signaling to the central nervous system (CNS) (Ma et al., 2022). Structural and functional disorders of the DRG, such as synaptic reorganization and alteration of voltage-gated sodium channels, are important for the progression of NeP. Considering the anatomical accessibility and functional specificity of DRG in NeP (Berger et al., 2021), further research on the pathogenesis of NeP at the level of the DRG would be of considerable significance to the development of novel treatments.

In recent years, there has been an increase in research on the regulation of NeP-related transcriptional genes. A transcription analysis of microRNAs (miRNAs), circular RNAs (circRNAs), and mRNAs in the DRG suggested that the circRNA-miRNA regulatory network is involved in paclitaxel-induced NeP (Mao et al., 2022). Other clinical and basic research has shown that circHIPK3 is highly abundant in serum from diabetes patients who suffered from NeP and in DRG from streptozocin-induced diabetic NeP rats (Wang et al., 2018). Advanced research has further applied single-cell transcriptomic analysis of somatosensory neurons in the DRG of spared nerve injury (SNI) rats, uncovering the temporal development of the NeP (Wang H. et al., 2021). These studies revealed that related miRNAs and circRNAs underlie the molecular mechanism of NeP genesis and development. However, the involvement of ceRNAs in the pathogenesis of NeP has not yet been studied in depth.

The purpose of this study was to explore the regulatory circRNA-miRNA-mRNA network in the DRG of mice induced by sciatic nerve transection (SNT). Finally, 2 circRNAs, 2 miRNAs and 10 mRNAs were used to construct a circRNA-miRNA-mRNA regulatory network, which may reveal new etiopathogenesis of NeP.

## 2. Materials and methods

### 2.1. Data source and DEGs definition

The public GSE96051 dataset was obtained from the Gene Expression Omnibus (GEO) database.^1^ The dataset contains 9 samples, including DRGs of sciatic nerve transection (SNT) mice (*n* = 5) and uninjured mice (Control) (*n* = 4). First, gene expression data quality was analyzed and visualized for each set of samples using the ggplot2 package of R software, which is an open-source software package for statistical computing and graphics.^2^ Then, differential expression genes (DEGs) analysis was performed for the “SNT” group versus the “Control” group with the use of the limma R package. |log2FC| > 1 and *P*-adjusted <0.05 were set as the cutoff criteria. Finally, the R language was utilized for result visualization. Heatmaps are graphical representations of data that represent each value as a color. Here, we used a heatmap to show the DEGs in NeP.

### 2.2. KEGG and GO enrichment analysis

The KEGG and GO functional enrichment analysis were performed using the online tool Metascape (Zhou et al., 2019). The GO analysis included analyses of biological processes, cellular components, and molecular functions. The top 20 enriched entries were displayed, and the results were visualized with the R language. The *p* value indicates the significance of the enriched entries under the corresponding conditions. The pathway is more significant if the *p* value is lower.

### 2.3. Protein–protein interaction network analysis and screening of hub genes

To evaluate the interrelationships among DEGs, we mapped the DEGs to String v11.5 (Szklarczyk et al., 2021). PPI networks were constructed using proteins encoded by the 421 differentially expressed mRNAs (DEmRNAs), which represent genes as nodes and interactions as lines. The disconnected nodes in the network were hidden. The key PPI network was screened using a plug-in, cytoHubba (Chin et al., 2014), in Cytoscape 3.9.0 software^3^ (Shannon et al., 2003). The top 10 nodes ranked by the MCC method were considered key PPI network nodes.

### 2.4. Construction of miRNA-mRNA network and functional annotation

The miRNAs potentially binding to the top 5 hub genes in the key PPI network were predicted by three databases (TargetScan, miRWalk, and miRDB) (Agarwal et al., 2015; Sticht et al., 2018; Chen and Wang, 2020). The total miRNAs were then enriched for function to screen for key miRNAs, and experimental validation was performed. The network connecting target genes and differentially expressed miRNAs was visualized using the Cytoscape 3.9.0 tool.

### 2.5. Construction of the circRNA-miRNA-mRNA ceRNA network

Considering the potential regulatory function of circRNAs in achieving the regulation of target gene expression by recruiting miRNAs, we predicted the potential targeting circRNAs of differentially expressed miRNAs using the online database starBase 3.0 (Li et al., 2014). The intersection of the prediction results of two key miRNAs was selected as key circRNA. Eventually, the circRNA-miRNA-mRNA network in NeP was established.

### 2.6. Animals

A total of 9 Adult male C57BL/6 mice (7–9 weeks old) were utilized in this study. Mice were caged at a temperature of 25 ± 1°C and followed a standard light/dark cycle of 12/12 h, with water and food available *ad libitum*. All procedures involving animals were approved by the Experimental Animal Welfare Ethics Committee of Fujian Medical University. All efforts were made to reduce suffering as well as the number of experimental animals. All investigators conducting the experiments were blinded to the grouping of the experimental animals.

### 2.7. Animal model of SNT

The SNT procedure was carried out as previously described (Hu et al., 2016). Briefly, mice were anesthetized using 1% pentobarbital sodium (50 mg/kg), and the right sciatic nerve was exposed at the mid-thigh level and sectioned distally. Once the modeling was complete, each layer opened was carefully closed with sutures. After three days, the ipsilateral L3 and L4 DRGs, as well as the contralateral uninjured DRGs as controls, were harvested for subsequent processing (Larhammar et al., 2017).

### 2.8. Behavioral tests

The paw mechanical withdrawal threshold (PMWT) was measured preoperatively and 3 days postoperatively. Mice were put in separate Plexiglas cells on a wire mesh floor and acclimated for 30 min prior to testing. Following the previous instructions (Dixon, 1980), Von Frey (Stoelting, Wood Dale, IL, United States) was applied to the ipsilateral and contralateral hind paw with the up-and-down method. The measurements of PMWT were undertaken according to a previously described approach (Chaplan et al., 1994). All behavioral studies were performed by an experimenter who was blinded to the group assignment.

### 2.9. Validation of the hub genes and miRNAs by quantitative real-time PCR

After the behavioral test, the mice were euthanized by decapitation under deep anesthesia on the third day after the operation. The L3/L4 DRGs were separated and immediately frozen in liquid nitrogen. A 10 mg tissue sample was mixed in TRIzol Reagent (Takara, Kusatsu, Japan) to isolate total RNA from tissues. A Primer Script RT reagent kit (TaKaRa, Code No. RR047A, No. 638313) was used to reverse transcribe the RNA into cDNA using a PCR instrument (2,720 Thermal Cycler, ThermoFisher), which was then subjected to qPCR using the TB Green^®^ Premix Ex Taq^™^ II (Tli RNase H Plus) and Mir-X TM miRNA First-Strand Synthesis Kit (TaKaRa, Code No. RR420A, No. RR820A) on a Roche LightCycler480 real-time PCR system. Five hub genes and two key miRNAs were selected for expression validation. Normalization of mRNA was achieved using GAPDH (Sangon, Shanghai, China) as an endogenous control gene, and U6 (Takara, No. 638313) was used as the internal reference gene of miRNA. The primer sequences were designed by Sangon Biotech (Sangon) and are listed in Table 1. The relative expression was calculated by the 2^−ΔΔCT^ method.

**Table 1:** Quantitative real-time polymerase chain reaction (RT-PCR) primer sequences.

Target	Sequence (5′ → 3′)
IL6	F: GGAGTCACAGAAGGAGTGGC
	R: AACGCACTAGGTTTGCCGAG
Jun	F: CCAAGAACGTGACCGACGAG
	R: GCGTGTTCTGGCTATGCAGT
Cd44	F: GGGTTTTGAAACATGCAGGTAT
	R: GTTGGACGTGACGAGGATATAT
Timp1	F: GCAAAGAGCTTTCTCAAAGACC
	R: CTCCAGTTTGCAAGGGATAGAT
Csf1	F: AACAGCTTTGCTAAGTGCTCTA
	R: ACTTCCACTTGTAGAACAGGAG
GAPDH	F: GTTACCAGGGCTGCCTTCTC
	R: GATGGTGATGGGTTTCCCGT
miR-223-3p	GCTGTCAGTTTGTCAAATACCCCA
miR-181a-5p	AACATTCAACGCTGTCGGTGAGT

### 2.10. Western blotting

On the 3rd day after SNT, the L3/L4 DRGs were collected and immediately frozen after the mice were sacrificed. The tissue was lysed in RIPA buffer containing phosphatase and protease inhibitors (Beyotime, Shanghai, China). A BCA protein assay kit (Beyotime) was used to determine the concentration of protein. The proteins were transferred to polyvinylidene fluoride membranes after gel electrophoresis and then incubated with primary antibodies at 4°C overnight after blocking with 5% nonfat dry milk in TBS + Tween. The primary antibodies were as follows: rabbit anti-IL-6 (1:1000, ab6672, Abcam), rabbit anti-c-Jun (1:1000, Cat. No. D151652, Sangon), rabbit anti-Cd44 (1:500, ab51037, Abcam), rabbit anti-Timp1 (1:1000, ab109125, Abcam), rabbit anti-Csf1 (1:1000, ab233387, Abcam), and β-actin rabbit mAb (1:1000, AC026, Abclonal, Wuhan, Hubei, China), and were processed with goat anti-rabbit IgG (1:4000, AS014, Abclonal) at room temperature for 1 h. The signals of target proteins and β-actin were detected by a chemiluminescence detection system (Tanon4600, Shanghai, China). Relative protein expression levels were normalized using β-actin as an internal reference, and the band intensity was analyzed using ImageJ software (National Institutes of Health, Bethesda, MD, United States).

### 2.11. Transmission electron microscopy analysis

On the 3rd day after SNT, the right injured and contralateral undamaged L3/L4 DRGs of mice were extracted. The samples were fixed in 2% glutaraldehyde, washed 3 times (15 min each time) in phosphate buffer (pH 7.4), postfixed in 1% osmium tetroxide in phosphate buffer (pH 7.4) for 2 h, and dehydrated under increasing alcohol concentrations. After embedding in epoxy resin medium, tissue was sliced into semithin sections (approximately 2 μm), stained with azure methylene blue, cut into ultrathin slices (approximately 60 nm), and stained with osmic acid. The morphology of the DRG neuron cell body and myelin were observed by transmission electron microscopy (HT7700, Hitachi, Japan). The ultrastructural assessment of nerve fiber myelin is consistent with the previously reported grading system (Table 2; Kaptanoglu et al., 2002). Ten myelinated axons in three samples per group were evaluated by this grading system. Besides, three views were randomly selected in each case, and the number of autophagosomes in the DRG was recorded for statistical analysis.

**Table 2:** Ultrastructural grading system of myelinated axons.

Score	Category
Grade 0	Normal
Grade 1	Separation in myelin configuration
Grade 2	Interruption in myelin configuration
Grade 3	Honeycomb appearance
Grade 4	Collapsed myelin-forming ovoids

### 2.12. Statistical analysis

Statistical analyses were performed using SPSS 21.0 (SPSS, Chicago, IL, United States) and GraphPad Prism 9 (GraphPad Software Inc., San Diego, CA, United States). Quantitative data are expressed as the mean ± SD. Student’s *t* test was used to analyze the comparison between two groups. The grade of myelin sheath damage was statistically analyzed using a chi-square test. A two-sided *p* value less than 0.05 was considered statistically significant.

## 3. Results

### 3.1. Identification of DEGs in NeP

**Figure 1:**
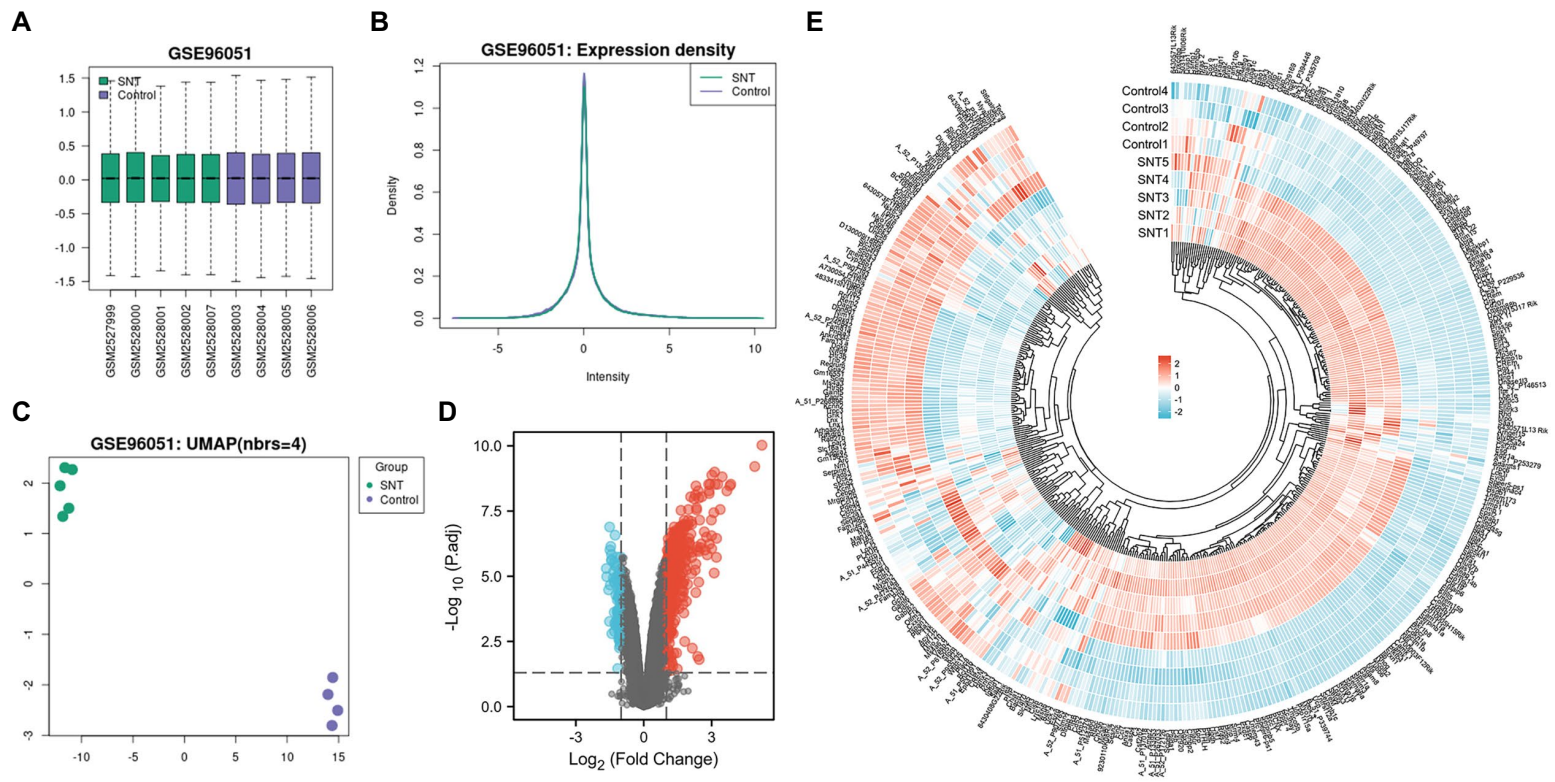
Differentially expressed profile of mRNAs and characterization between the SNT group and the control. **(A)** Boxplot shows the distribution of the values of the DRG samples, abscissa represents sample name and ordinate represents the normalized intensity values, the upper and lower sides of the rectangular box mean minimum and maximum values, the upper and lower lines of the error bars mean interquartile range, the line inside the rectangular box means median value. **(B)** The density map of GSE96051 shows the expression of each sample. **(C)** The UMAP shows the distribution relationships between the samples. **(D)** Volcano plot of DEmRNAs. The vertical lines correspond to twice the fold changes in upregulation or downregulation; the horizontal line represents *p*-adjusted = 0.05; red points indicate mRNAs with upregulation and blue points indicate mRNAs with downregulation. **(E)** Hierarchical cluster analysis of DEmRNAs. Blue and red color indicate down-regulation and up-regulation of gene expression, respectively.

The boxplot automatically generated by GEO shows the normalized data, and the distribution of values in the GSE96051 dataset is relatively consistent across all samples, indicating that the location and dispersion of the data meet the quality requirements (Figure 1A). Samples from the SNT group displayed a green color, and samples from the control group displayed a purple color. The expression density plots complement the boxplot in examining data normalization by visually depicting the distribution of gene expression in each sample before performing DEGs analysis. The intensity of genes was mainly between-2 and 2 with little variation between samples, indicating their consistency in respective datasets and ideal sample data quality (Figure 1B). Uniform manifold approximation and projection (UMAP) is a dimensionality reduction technique that can be used to visualize how samples are related to each other. The distance between points (sample to sample) can reflect the obvious difference between groups and the consistency within the group (Figure 1C). The volcano map shows DEGs in the two groups, with upregulated genes and downregulated genes marked in red and blue, respectively (Figure 1D). These DEGs are displayed in Supplementary Table S1. Heatmap shows hierarchical cluster analysis of DEmRNAs, including 332 upregulated genes and 89 downregulated genes. The color of genes from blue to red indicates a low or high level of gene expression (Figure 1E).

### 3.2. KEGG and GO enrichment analyses

**Figure 2:**
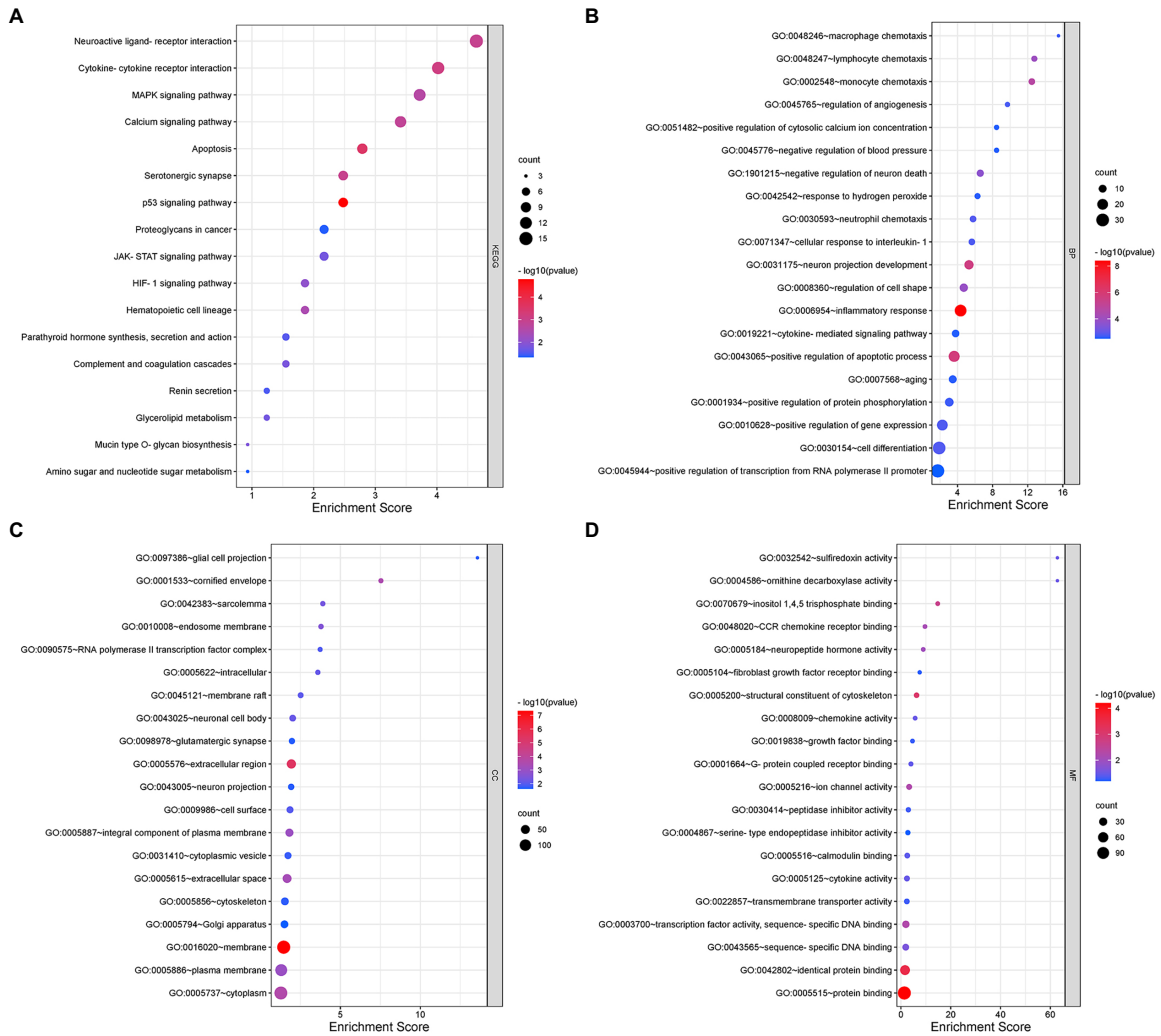
Gene Ontology (GO) and Kyoto Encyclopedia of Genes and Genomes (KEGG) analyses for DEmRNAs. **(A)** KEGG pathway analysis shows the significantly enriched pathways and their gene counts. **(B–D)** The GO enrichment results of DEmRNAs, including GO-BP **(B)**, GO-CC **(C)**, and GO-MF **(D)**.

KEGG functional analysis of DEmRNAs showed the top 17 representative enrichment pathways, including the MAPK signaling pathway, calcium signaling pathway, p53 signaling pathway, HIF-1 signaling pathway, and cytokine-cytokine receptor interaction (Figure 2A; Supplementary Table S2).

Furthermore, we performed GO analysis of DEGs, and the enriched results were highly significant, including 131 entries enriched in biological process (BP), 36 entries in cellular component (CC) and 34 entries in molecular function (MF) (Figures 2B–D; Supplementary Table S2). For BP, DEmRNAs were mainly enriched in inflammatory and immune processes as well as endocrine metabolism processes. The inflammatory and immune processes included inflammatory response (GO:0006954), neutrophil chemotaxis (GO:0030593), cellular response to interleukin-1 (GO:0071347), macrophage chemotaxis (GO:0048246), cytokine-mediated signaling pathway (GO:0019221), positive regulation of tumor necrosis factor production (GO:0032760), immune system process (GO:0002376), etc. The metabolic processes included positive regulation of apoptotic process (GO:0043065), negative regulation of neuron death (GO:1901215), regulation of angiogenesis (GO:0045765), positive regulation of calcium ion import (GO:0090280), positive regulation of receptor-mediated endocytosis (GO:0048260), positive regulation of serotonin secretion (GO:0014064), positive regulation of chemokine production (GO:0032722), etc. For CC, almost all of the enriched terms were related to cellular metabolic processes, intracellular organelles and signal transmission, such as neuronal cell body (GO:0043025), glial cell projection (GO:0097386), neuron projection (GO:0043005), glutamatergic synapse (GO:0098978), Golgi apparatus (GO:0005794), axon cytoplasm (GO:1904115), and postsynaptic density (GO:0014069). Regarding MF, the enriched terms were almost all associated with protein binding, sequence-specific binding and ion channel activity, including chemokine receptor binding (GO:0048020), neuropeptide hormone activity (GO:0005184), calmodulin binding (GO:0005516), transcription factor activity (GO:0003700), sequence-specific DNA binding (GO:0043565), transmembrane transporter activity (GO:0022857), extracellular-glutamate-gated ion channel activity (GO:0005234), and ligand-gated ion channel activity (GO:0015276).

In summary, the involvement of intracellular and extracellular signaling pathways in immune, inflammatory and oxidative stress processes as well as endocrine metabolic processes may be closely related to the onset and development of NeP.

### 3.3. Establishment of the protein–protein interaction network and identification of hub genes

**Figure 3:**
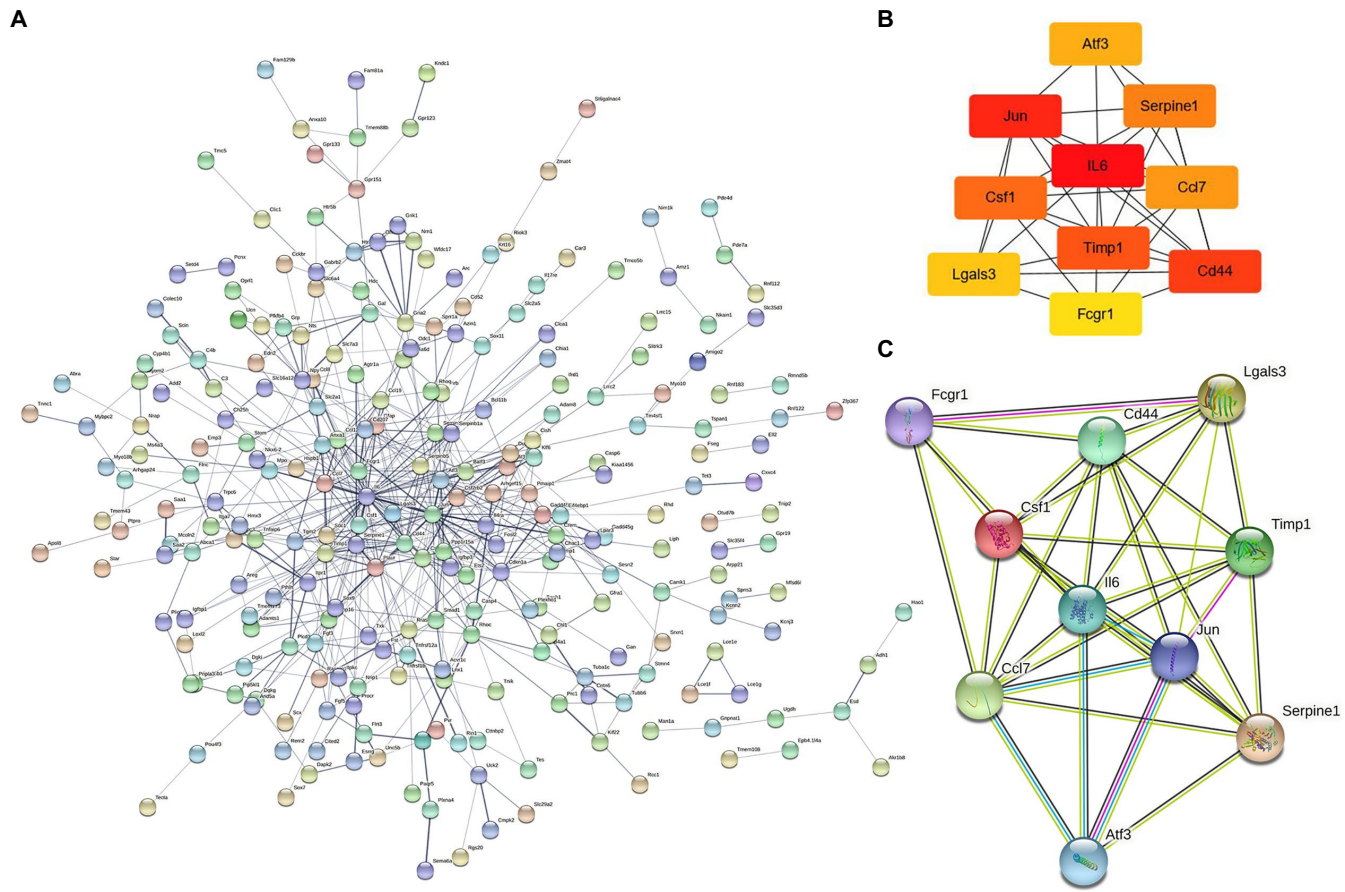
The construction of protein–protein interaction network and identification of hub genes. **(A)** PPI network is established by 421 DEmRNA-encoded proteins using the STRING website. **(B)** Relationship network diagram of hub genes from PPI network using cytoscape. **(C)** Relationship network of 10 hub genes that extracted from the PPI network and the structure of proteins they encoded.

The 421 DEGs (log2FC > 1) in NeP form a PPI network (Figure 3A) based on the STRING database, including 332 upregulated genes and 89 downregulated genes. The critical PPI network was established using Cytoscape, and the top 10 nodes ranked by the MCC algorithm were screened as hub genes: IL6, Jun, Cd44, Timp1, Csf1, Serpine1, Ccl7, Atf3, Lgals3, and Fcgr1 (Figure 3B). Figure 3C presents the interaction relationships of the 10 hub genes in the STRING database and the structures of the proteins they encode. Given that highly connected hub nodes have important functions in biological networks, the 10 hub genes were likely to play important roles in NeP, and the top 5 hub genes were selected for subsequent analysis and validation.

### 3.4. Western blot analyses of hub genes in the PPI network

**Figure 4:**
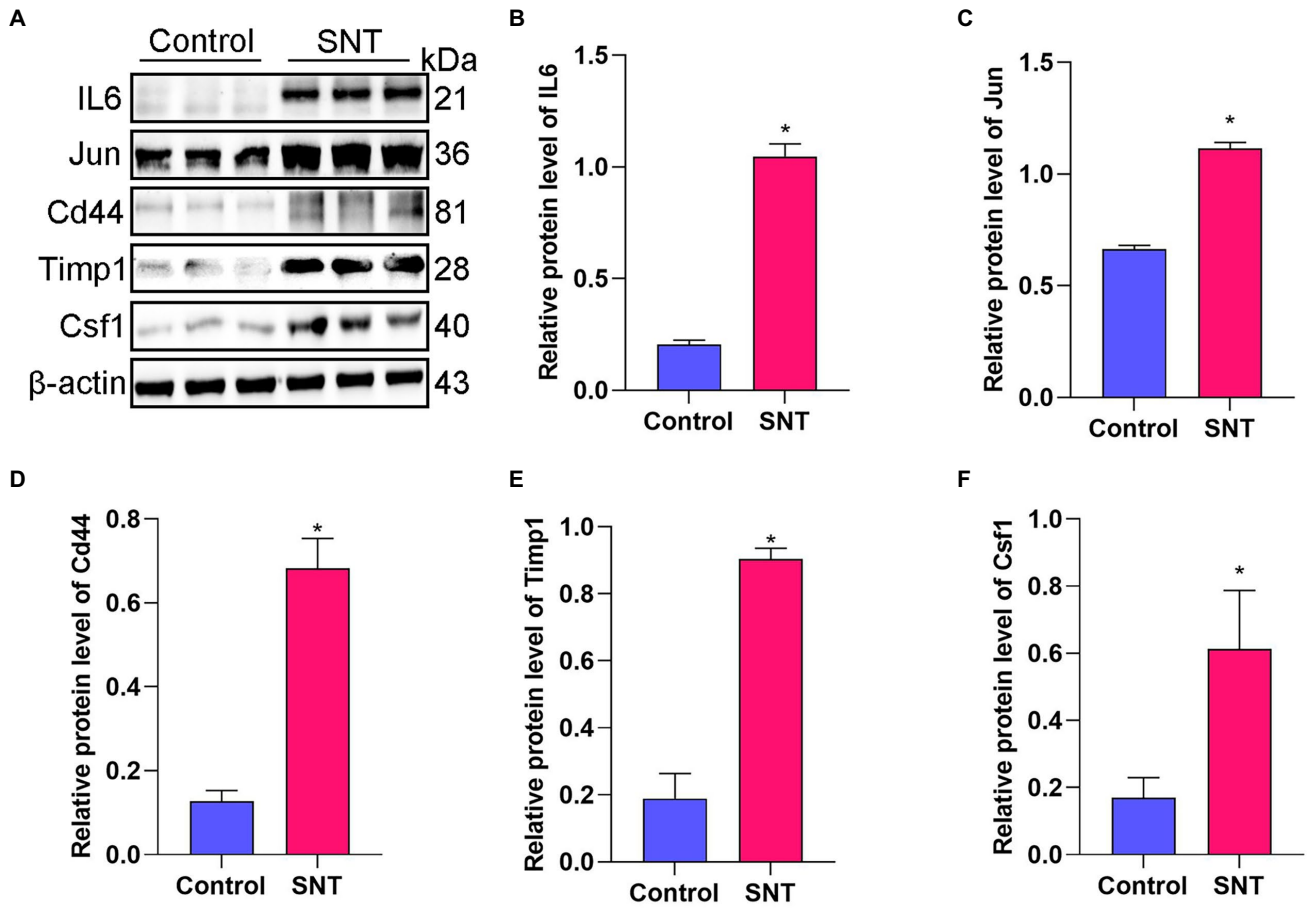
Verification of the top 5 hub genes. **(A)** The expression of the IL6, Jun, Cd44, Timp1 and Csf1 in DRG tissues of mice induced by SNT and the control by Western blot. **(B–F)** The expression analysis of the IL6, Jun, Cd44, Timp1 and Csf1. Data are presented as means ± SD (*n* = 3; *p < 0.05).

The top five DEGs in the PPI network were selected to check their expression levels by Western blot, including IL-6, Jun, Cd44, Timp1, and Csf1. The results showed that the protein expression levels of IL-6, Jun, Csf1, Timp1, and Cd44 in the SNT group were significantly upregulated compared with those in the control group (all *p* < 0.05) (Figure 4). The result of Western blot is broadly in line with that of sequencing.

### 3.5. Construction of the miRNA-mRNA-binding protein network and biological functional analysis

**Figure 5:**
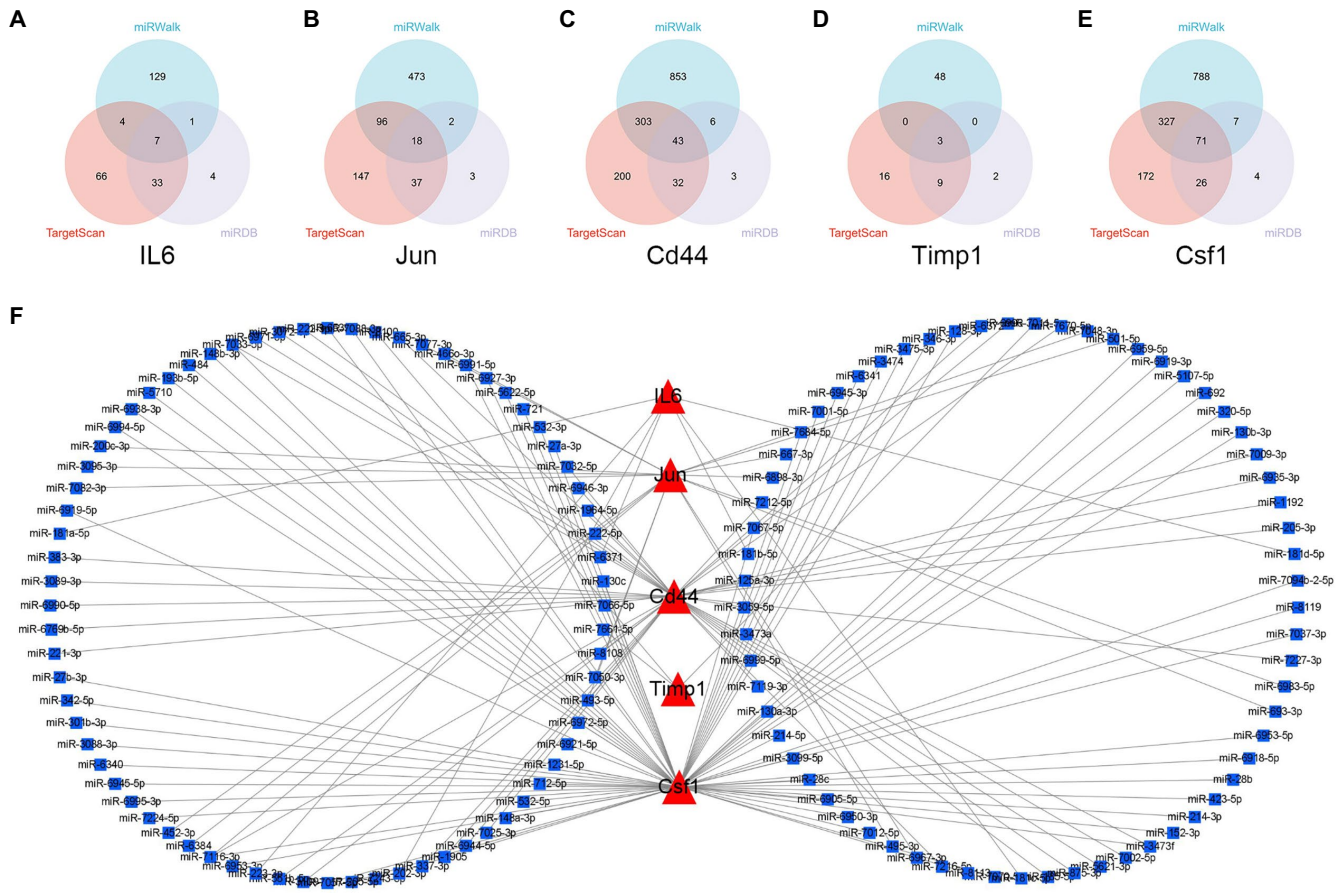
Construction of miRNA-mRNA network. **(A–E)** Overlap analysis for the miRNAs binding to the IL6, Jun, Cd44, Timp1, and Csf1 from the TargetScan, miRWalk, and miRDB database. **(F)** The miRNAs-hub genes network.

**Figure 6:**
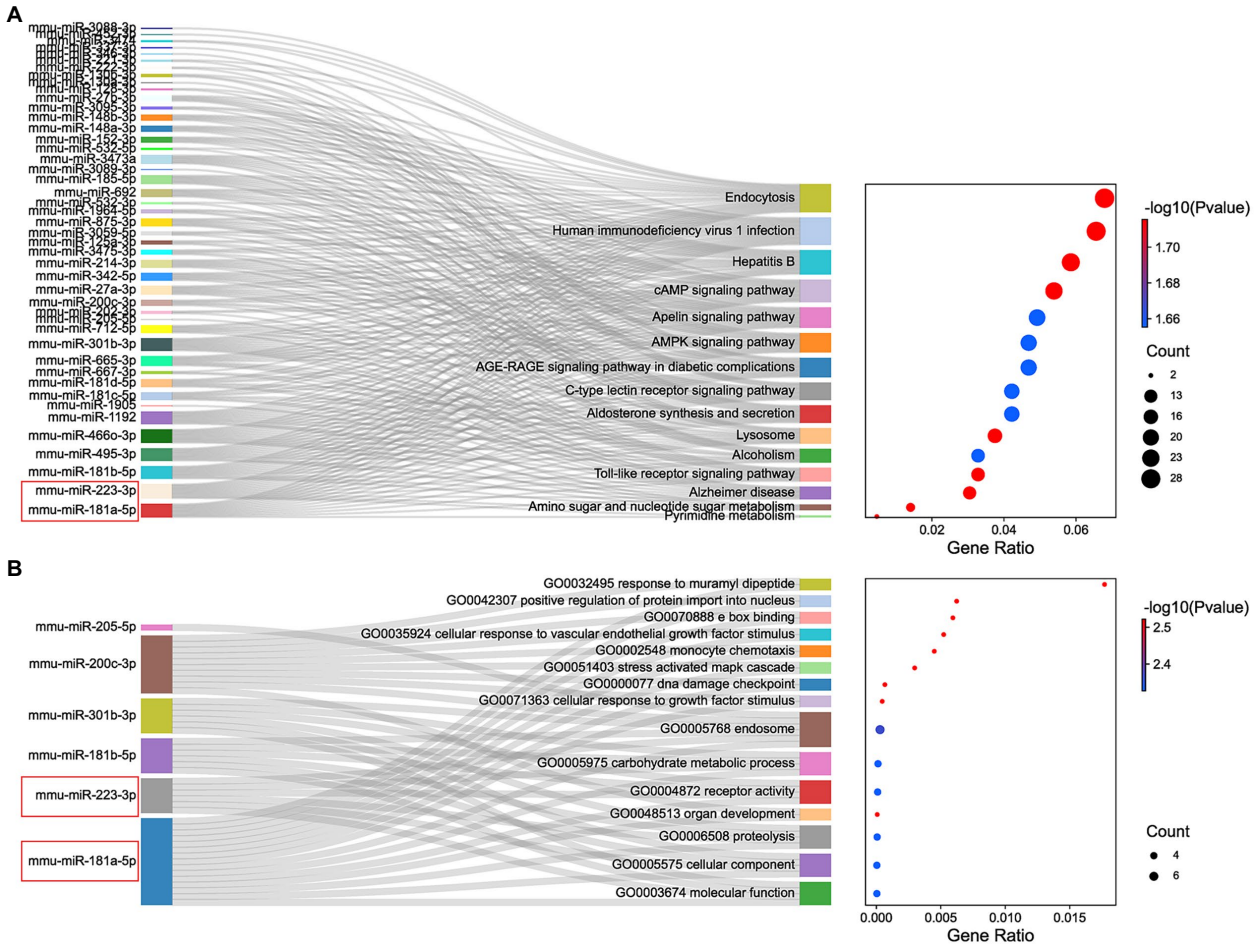
Functional enrichment analysis of miRNAs. **(A)** KEGG pathways enriched by the shared miRNAs between 3 different databases, TargetScan, miRWalk, and miRDB. **(B)** The GO enrichment analysis shows the top 15 significant items and their gene counts.

The miRNAs binding to the top 5 hub genes were predicted by three public databases (TargetScan, miRWalk and miRDB), and the intersections were used to determine the key miRNAs (Figure 5). In total, 7, 18, 43, 3, and 71 target miRNAs for IL6, Jun, Cd44, Timp1, and Csf1, respectively, were predicted (Figure 5; Supplementary Table S3). The miRNA Enrichment Analysis and Annotation Tool (miEAA) contributes to the functional analysis of target miRNAs (Kern et al., 2020). The results of KEGG analysis showed that these miRNAs were primarily enriched in pathways related to immune inflammation, oxidative stress, endocrine metabolism and neural signaling, including the chemokine signaling pathway, Toll-like receptor signaling pathway, IL-17 signaling pathway, MAPK signaling pathway, neurotrophin signaling pathway, long-term potentiation and long-term depression. Notably, numerous autophagy-related entries were enriched, such as autophagy, endocytosis, lysosome, phagosome, and protein processing in endoplasmic reticulum (Figure 6A; Supplementary Table S4). Additionally, the results of GO functional enrichment indicate that these miRNAs are involved in the cyclic nucleotide biosynthetic process (GO0009190), mRNA binding (GO0003729), protein complex assembly (GO0006461), regulation of dopamine secretion (GO0014059), regulation of myelination (GO0031641), and regulation of axonogenesis (GO0050770), especially in the autophagic vacuole (GO0005776), phagocytosis (GO0006909), cytoplasmic vesicle membrane (GO0030659), and endoplasmic reticulum Golgi intermediate compartment (GO0005793) (Figure 6B; Supplementary Table S5).

In summary, the strong correlation between the KEGG pathway and GO enrichment analysis suggests that potential DEmRNA-miRNA interactions may play a role in the development of NeP. Based on the remarkable prominence of mmu-miR-181a-5p and mmu-miR-223-3p in the functional enrichment results, we selected them for subsequent analysis and verification.

### 3.6. Changes in pain threshold and DRG ultrastructure after SNT

**Figure 7:**
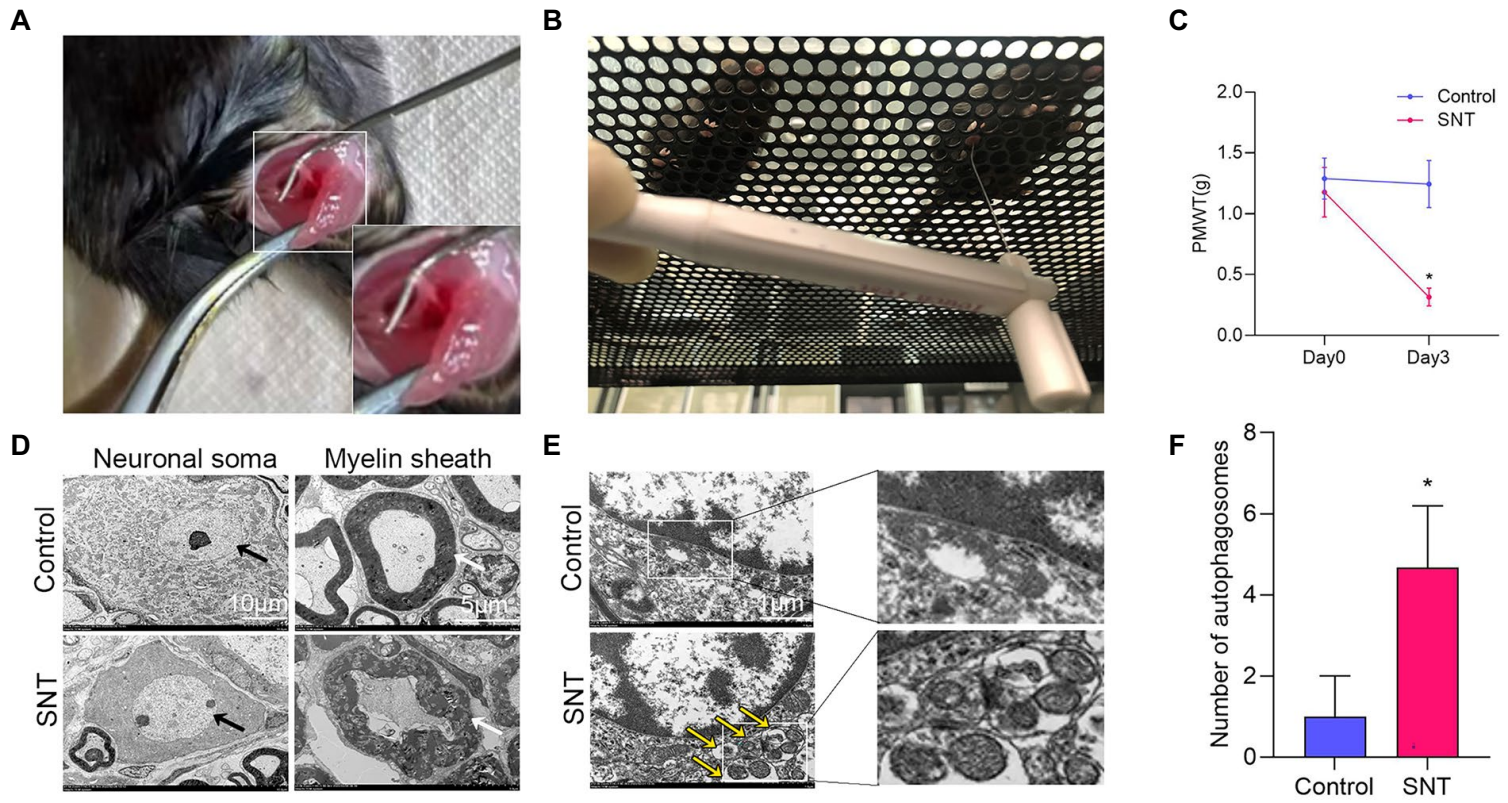
Changes of pain threshold and DRG ultrastructure after SNT. **(A)** The right sciatic nerve of mice was exposed and transected. **(B)** Behavioral test of mice using Von Frey. **(C)** SNT induced reduction of PMWT in mice. **(D)** Electron microscope analysis of the ultrastructural changes in L3/L4 DRGs on the 3rd day after SNT in different groups. The morphology of neuronal somata and myelin sheaths of DRG neurons in two groups. The neuron nucleus is indicated by the black arrow, Scale bar = 10 μm; magnification ×1,000; The myelin sheath is indicated by the white arrow, Scale bar = 5 μm; magnification ×2,000. **(E)** Representative electron microscope views of autophagosomes (yellow arrow) in DRG of the two groups. Scale bar = 1 μm; magnification ×12,000. **(F)** The number of autophagosomes of the two groups in per vision was determined on the 3rd day after SNT. Data are presented as means ±SD (*n* = 3; *p < 0.05).

As shown in the illustration (Figure 7A), the right sciatic nerve of the mice was exposed and cut off, resulting in hyperalgesia (Zhang et al., 2021). The baseline PMWT was measured preoperatively and at 3 days after the surgery (Figure 7B). Compared with the contralateral, ipsilateral hind paw showed a significantly lower pain threshold at 3 days after surgery (*p* < 0.05) (Figure 7C). The results suggested that mice undergoing SNT surgery exhibited NeP behaviors.

Further observation of the ultrastructure of the myelin sheath and cell body by electron microscopy revealed that SNT induced degeneration of a proportion of nerve fibers in the DRG. Compared to those of the control, myelin damage grading scores were significantly higher in the SNT group (Table 3) (*p* < 0.05), accompanied by structural abnormalities of the cell body (Figure 7D). In particular, DRG neuronal soma was regular with homogeneous cytoplasm and uniform and loose chromatin in the control group. The myelin sheath of nerve fibers was arranged in concentric circles with distinct layers. In the SNT group, the soma and myelin sheath of DRG were severely damaged with nerve demyelination, and partial myelin lamellar structures were completely destroyed on the 3rd day after SNT. Specifically, the neuronal soma displayed cytoplasmic shrinkage or swelling, nuclear pyknosis and displacement, and heterochromatin aggregation, and the membrane of cells was obscure with slightly enlarged space. The nerve medullary sheath had an unclear layer structure characterized by axis cylinder destruction even with a honeycomb-like appearance and fractured loose layers. The above results suggested that SNT does not cause axonal degeneration in the contralateral DRG, whereas it induces axonal injury in the ipsilateral DRG.

**Table 3:** Ultrastructural grading scores of all groups on the 3rd day after SNT.

Score	Control	SNT
Grade 0	21	3
Grade 1	9	5
Grade 2	0	8
Grade 3	0	10
Grade 4	0	4

Considering that in addition to axon-related entries, autophagy-related entries were also abundantly enriched in GO and KEGG, we further observed alterations in DRG autophagy after SNT by electron microscopy and discovered that the number of autophagosomes in DRG was increased significantly in the SNT group compared to the control group (Figures 7E,F) (*p* < 0.05).

These results not only suggest that the biological process of autophagy and DRG ultrastructural damage are involved in NeP but also illustrate the accuracy of the analysis at the ultrastructural level.

### 3.7. Confirmation of differentially expressed mRNAs and miRNAs by qRT-PCR

**Figure 8:**
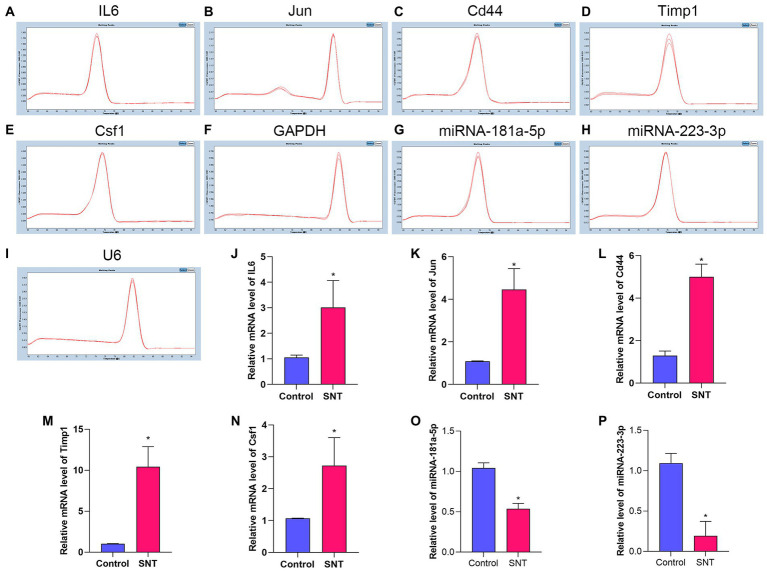
Amplification and identification of differentially expressed mRNAs and miRNAs. **(A–I)** The melt curves of the identified mRNAs and miRNAs. **(J–P)** The results of qRT-PCR show the expression levels of DEGs between the two groups. Data are presented as means ± SD (*n* = 3; *p < 0.05).

The top 5 hub genes and 2 key miRNAs were selected to conduct qRT-PCR on independent samples to check the reliability of the RNA-sequencing data (*n* = 3 per group, Figure 8). The results showed that the expression of both targeting miRNAs was significantly lower in the SNT group than in the control group (Figure 8) (all *p* < 0.05). The quantitative PCR findings matched the results of RNA sequencing (Table 4).

**Table 4:** The comparison information of the RNA-Seq and qRT-PCR data of the SNT mice vs. the control.

Gene name	RNA-Seq		qRT-PCR	
	Log_2_ fold change	*p* value	Fold change	*p*-value
IL6	2.334745	0.0000000287	3.010896	0.0334
Jun	1.769795	0.000256	4.459487	0.0039
Cd44	1.0585	0.0000431	4.999413	0.0005
Timp1	1.117385	0.000008	10.432013	0.0026
Csf1	2.00039	0.000000353	3.454678	0.0310

### 3.8. Prediction of differentially expressed miRNAs

**Figure 9:**
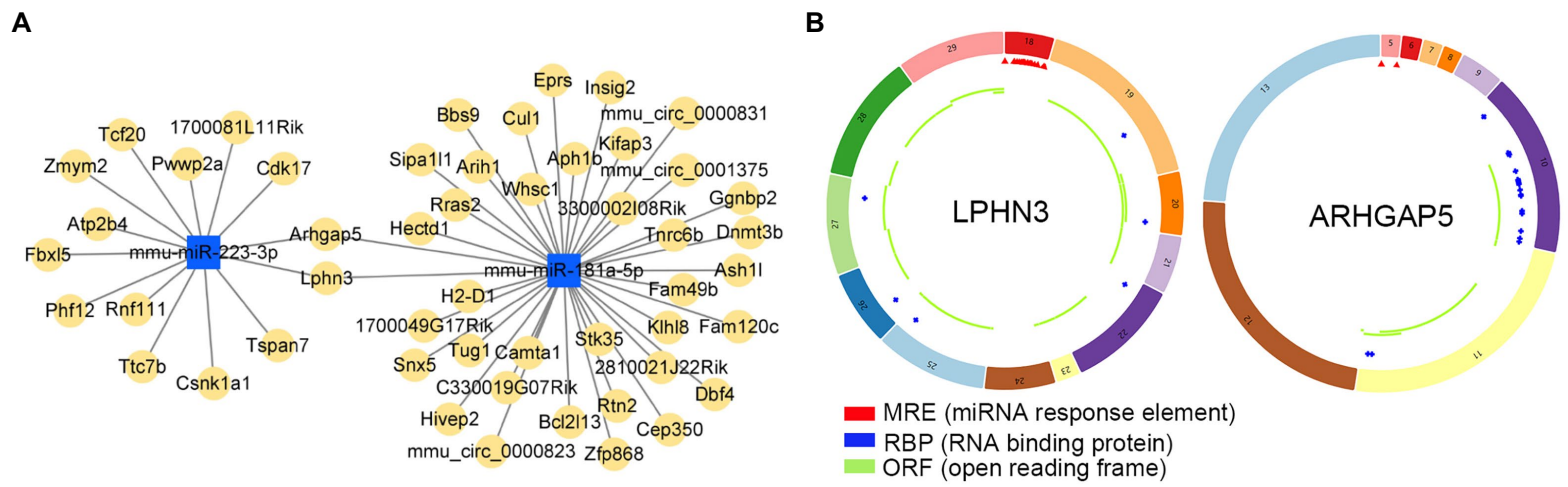
The analysis of circRNA-miRNA networks. **(A)** The competing endogenous RNA networks of DEmiRNAs. **(B)** Structural patterns of circLPHN3 and circARHGAP5. The colored circle represents the circRNAs that consist of exons. The numbers on the circRNAs mean the exon number. The red,blue, and green regions inside the circRNA molecule, respectively represent MRE (microRNA response element), RBP (RNA binding protein), and ORF (open reading frame).

Potential target circRNAs were identified based on predictive analysis of circRNA-miRNA interactions, and relevant ceRNA regulatory networks were constructed (Figure 9A; Supplementary Table S6). Noticeably, the prediction results showed that circLPHN3 and circARHGAP5 could target key DEmiRNAs mmu-miR-181a-5p and mmu-miR-223-3p. Accordingly, circLPHN3 and circARHGAP5 were identified as important circRNAs. The structural pattern graphs of the 2 significant circRNAs in the ceRNA network were drawn by the CSCD database (Xia et al., 2018), and they can be used to predict miRNA response elements, RNA binding proteins, and open reading frames to further investigate the potential functions of the circRNAs (Figure 9B).

### 3.9. Construction of the circRNA-miRNA-mRNA ceRNA network

**Figure 10:**
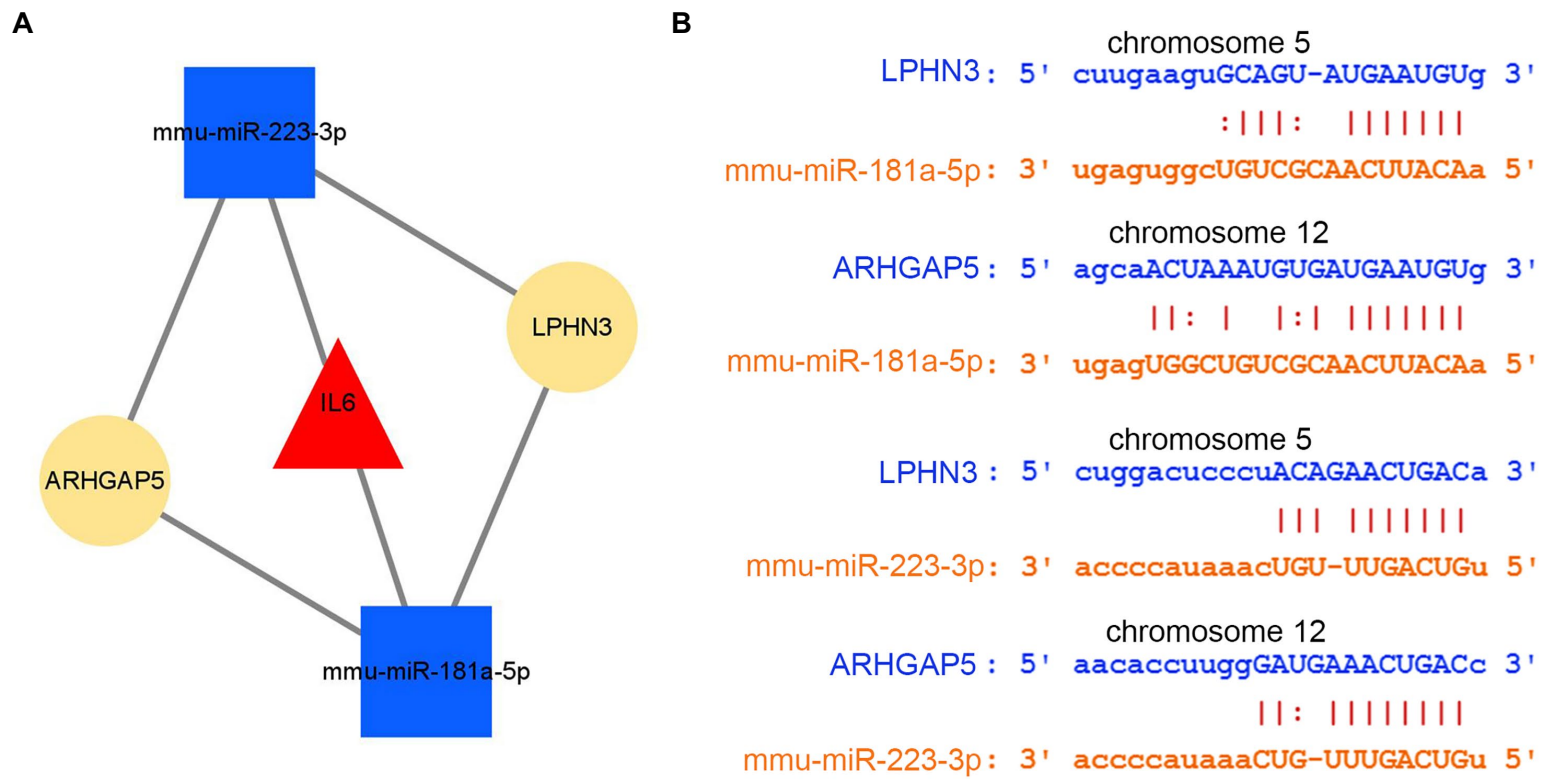
The analysis of circRNA-miRNA-mRNA network. **(A)** The circRNA–miRNA-hub gene network. **(B)** The interaction of novel circRNAs/DEmiRNAs.

By intersecting the predicted results of two miRNAs, mmu-miR-181a-5p and mmu-miR-223-3p, both circLPHN3 and circARHGAP5 were found to be able to target the two miRNAs and they were identified as essential circRNAs accordingly. Among the 5 DEmRNAs, only IL6 was predicted by the databases to target both mmu-miR-181a-5p and mmu-miR-223-3p. As a result, 2 miRNAs target a key DEmRNA (IL6), and 2 circRNAs further target 2 DEmiRNAs, forming a circRNA-miRNA-mRNA network (Figure 10A). The binding sequence between target circRNAs and miRNAs was obtained by downloading from the starBase3.0 database (Figure 10B).

## 4. Discussion

As a widespread chronic disease, NeP has a complex clinical presentation and a long course and is often combined with sleep disorders, anxiety and depression. NeP severely reduces the quality life of patients and imposes a significant economic burden on society (Wu et al., 2019). The exact mechanism underlying the role of ceRNAs in NeP is not yet clear. Analysis of the circRNA-miRNA-mRNA expression profile may provide new insights into the pathophysiology of NeP. In previous studies, several research groups have identified dysregulated miRNAs or long noncoding RNAs (lncRNAs) in the DRGs of mice with different NeP models using deep RNA-seq analysis (Mao et al., 2018; Jia et al., 2020). However, little is known about the systematic study of circRNAs, especially the role of the circRNA-miRNA-mRNA regulatory network in NeP.

At present, the DRG is considered to be a key structure in sensory transmission and modulation, including pain transmission and maintenance of a persistent neuropathic pain state. The unique properties of the DRG, including selective somatic cell organization, specific membrane properties and an easily accessible and consistent location, make it an ideal target for neuromodulation (Esposito et al., 2019). In this study, we used bioinformatics to identify potential DRG biomarkers for NeP by constructing related ceRNA networks to explore their possible molecular regulatory mechanisms. We identified 421 DEmRNAs (log2FC > 1) and 2 miRNAs (mmu-miR-181a-5p and mmu-miR-223-3p) in response to SNT model building. A few studies have reported the involvement of miR-181a-5p in several neurological disorders. Mechanistically, miR-181a-5p inhibition regulates cell survival in neurons and astrocytes after forebrain ischemia and stroke (Arvola et al., 2019), and lncRNA SNHG1 promotes neuronal injury in a Parkinson’s disease cell model *via* the miR-181a-5p/CXCL12 axis (Wang K. et al., 2021). However, the involvement of miR-181a-5p in the pathogenesis of NeP has not yet been reported. Encouragingly, limited research has preliminarily revealed the involvement of miR-223-3p in NeP. For example, trigeminal NeP can be alleviated by miR-223-3p targeting MKNK2 and MAPK/ERK signals in male mice (Huang et al., 2022). Another study also reported that electroacupuncture inhibits autophagy in neuronal cells by increasing the expression of miR-223-3p in postherpetic neuralgia (Zou et al., 2021). In addition, one clinical study identified that miR-223-3p in cerebrospinal fluid was significantly lower in fibromyalgia patients than in healthy controls (Bjersing et al., 2013). Notably, the analysis of this study revealed that the target miRNAs and IL6 and their strong correlations may play a role in NeP, which is consistent with the results of a previous study (Hori et al., 2016). For example, intrathecal injection of miR-214-3p can reverse enhanced CSF1 expression and astrocyte overactivity and alleviate the IL-6 upregulation and pain behavior in in rats with spinal nerve ligation (Liu et al., 2020). Besides, another key DEG, Jun, is an oncogene that can activate the cAMP pathway, has been demonstrated that Jun complex promoted the progression of NeP *via* JNK pathway (Xu C. et al., 2022). These results suggest that the miRNA-mRNA network may play an important regulatory role in NeP.

CircRNAs are a type of noncoding, regulatory RNAs that exhibit tissue-specific and disease-specific expression. An increasing number of studies have reported that circRNAs may play pivotal roles in the development of NeP (Xu D. et al., 2021). Therefore, we further constructed circRNA-miRNA ceRNA networks to demonstrate their interactions, and circRNAs (circLPHN3 and circARHGAP5) predicted by both mmu-miR-181a-5p and mmu-miR-223-3p may play an essential role in the regulatory networks. Here, we suggest that the role and function of circRNAs as ceRNAs in the DRG of the NeP model are worth further investigation.

KEGG and GO analysis based on the above DEmRNAs and corresponding potential binding miRNAs showed similar results. The enriched KEGG pathways were related to immune inflammation, oxidative stress, endocrine metabolism and neural signaling. Immune inflammation reactions (Hu et al., 2020), oxidative stress (Xu J. et al., 2021), genomic metabolic analysis (Xu Z. et al., 2022) and neural signaling (Chen et al., 2021) in the DRG have previously been associated with pathogenesis in NeP. Similar to KEGG, the results of the GO analysis focused on cellular processes and bioregulation, including regulation of axonogenesis (GO0050770), autophagic vacuole (GO0005776), nucleotide biosynthetic process (GO0009190), mRNA binding (GO0003729), ligand-gated ion channel activity (GO:0015276), etc. These results further confirm the regulatory role of miRNA-mRNA networks in NeP.

Considering that the above DEmRNAs and corresponding potential binding miRNAs for KEGG and GO functional analysis were abundantly and significantly enriched in the entries related to autophagy and axons. We further observed the changes in DRG ultrastructure and autophagosomes after SNT by electron microscopy. The results imply that the SNT could induce a certain degree of nerve fiber demyelination (Dun and Parkinson, 2018), which not only demonstrates the accuracy of functional enrichment analysis but also provides credibility to the modeling. In addition, behavioral observation is consistent with a previous study showing that the SNT induces mechanical hyperalgesia (Manassero et al., 2012). Autophagy has been proved to participate in various biological processes of diseases, including NeP. In recent years, increasing evidence has shown that ceRNAs influence the course of a disease by regulating many genes involved in autophagy, suggesting that autophagy is involved in the onset and progression of various diseases and can affect drug resistance (Cai W. et al., 2020; Cai L. et al., 2020; Zou et al., 2021; Wang et al., 2022). In this study, a significant increase in the number of autophagosomes was observed in the SNT-induced NeP mice, which may indicate that the mechanism of ceRNA involvement in NeP may be associated with the autophagic pathway.

Most of the previous studies on the mechanism of NeP were based on animal models, however, these studies did not systematically describe the changes in DRG that occur in NeP, which may be an important obstacle for DRG-related treatment and research. To the best of our knowledge, this is one of the few studies to reveal the potential mechanism of NeP by integrating the analysis of mRNA, miRNA and circRNA in DRG. The circRNA-miRNA-mRNA regulatory network constructed in this study will contribute to further understanding of the involvement of DRG in the pathogenesis of NeP. This study still has some limitations. First, we only used the sequencing results of 1 dataset due to limited data, and it may be possible to reduce the variation in sequencing results and make the analysis more convincing if multiple sequencing results were applied and intersected. Second, we only observed the SNT as a NeP model, and whether there are inconsistencies in the ceRNA regulatory network for different model conditions needs further investigation. Finally, the expression level of circRNA was not determined, and the regulatory analysis of the ceRNA network can be further verified and comprehensively explored by experimental methods such as gene overexpression, gene knock-out and dual-luciferase reporter assays in the future.

In conclusion, we constructed a ceRNA network associated with miRNAs and circRNAs to identify potential mechanisms of NeP. Our findings suggest that specific miRNAs and circRNAs may help explore candidate targets and new molecular biomarkers for NeP therapy. The results of this study provide preliminary confirmation that the novel circLPHN3/circARHGAP5_mmu-miR-223-3p/mmu-miR-181a-5p_IL6 networks may regulate the pathophysiology of NeP by affecting multiple signaling pathways. These newly identified networks and genes in the signaling pathway reveal potential diagnostic and therapeutic targets for NeP. However, whether these associations contribute to the development of NeP remains to be further studied.

## Data availability statement

Publicly available datasets were analyzed in this study. This data can be found at: https://www.ncbi.nlm.nih.gov/gds, and accession number is GSE96051.

## Ethics statement

The animal study was reviewed and approved by the Animal Ethics Committee of Fujian Medical University.

## Author contributions

YY and XX completed the experiments, analyzed the data, and wrote the manuscript. RL conceived the study, obtained funding, and critically revised the manuscript. CL offered lab instruments, participated in data analysis, and the revised version. All authors read and approved the final manuscript.

## Funding

This work was supported by the Project of Medical Innovation of Fujian Province, China (2018-CX-6) and the Startup Fund for Scientific Research of Fujian Medical University, China (2019QH1151).

## Conflict of interest

The authors declare that the research was conducted in the absence of any commercial or financial relationships that could be construed as a potential conflict of interest.

## Publisher’s note

All claims expressed in this article are solely those of the authors and do not necessarily represent those of their affiliated organizations, or those of the publisher, the editors and the reviewers. Any product that may be evaluated in this article, or claim that may be made by its manufacturer, is not guaranteed or endorsed by the publisher.

## Footnotes

^1^ www.ncbi.nlm.nih.gov/geo/

^2^ http://www.bioconductor.org/

^3^ https://cytoscape.org/
