# Supplementary figures and images for: Systematic identification of potential key microRNAs and circRNAs in the dorsal root ganglia of mice with sciatic nerve injury
Source: Front Mol Neurosci. 2023 Mar 14;16:1119164. doi: 10.3389/fnmol.2023.1119164 (PMC10043392; doi:10.3389/fnmol.2023.1119164)

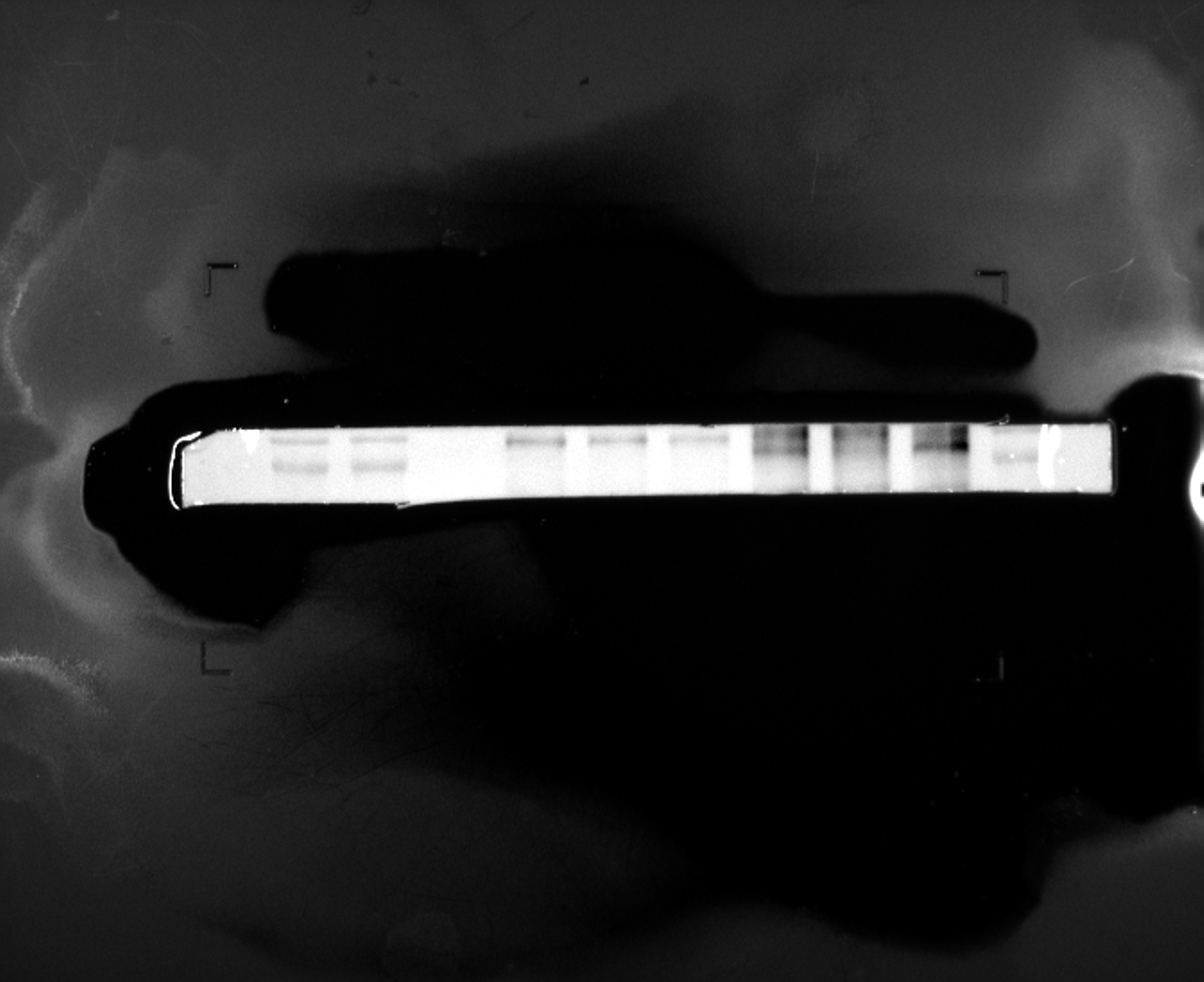

Supplement: Supplementary file 8 [file Data_Sheet_2.ZIP › Scans of the original gels/CD44.Tif]

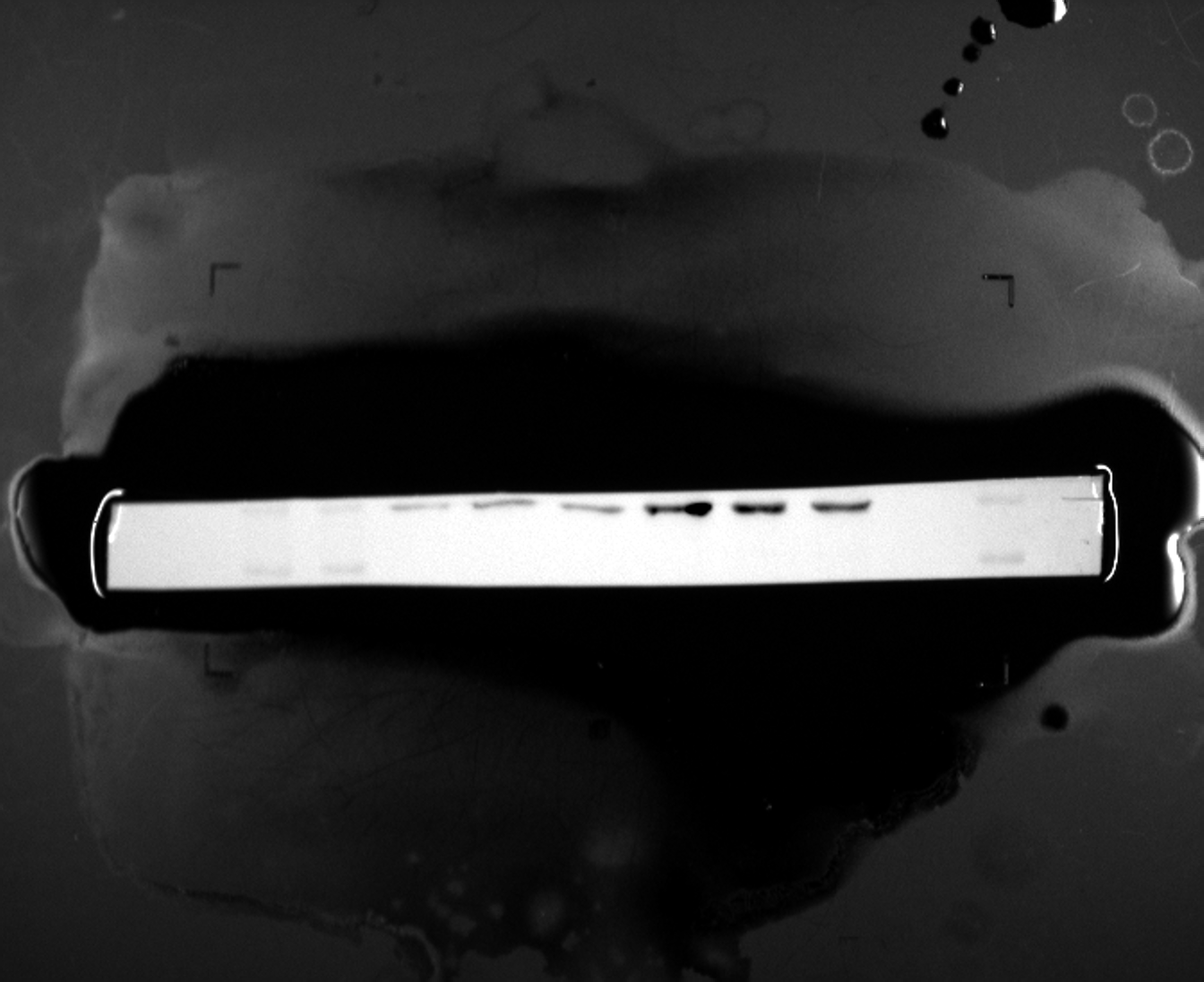

Supplement: Supplementary file 8 [file Data_Sheet_2.ZIP › Scans of the original gels/CSF1.Tif]

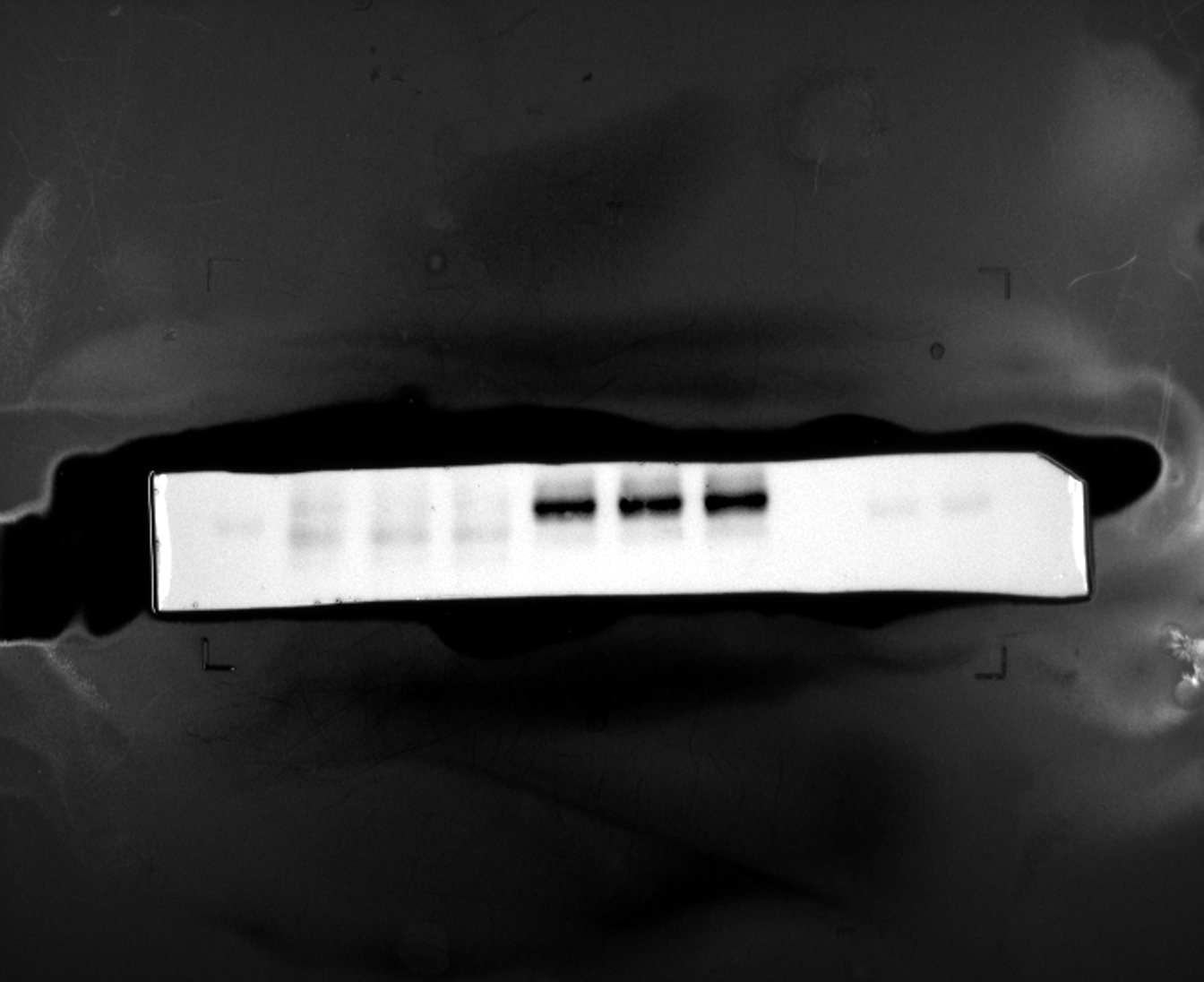

Supplement: Supplementary file 8 [file Data_Sheet_2.ZIP › Scans of the original gels/IL6.Tif]

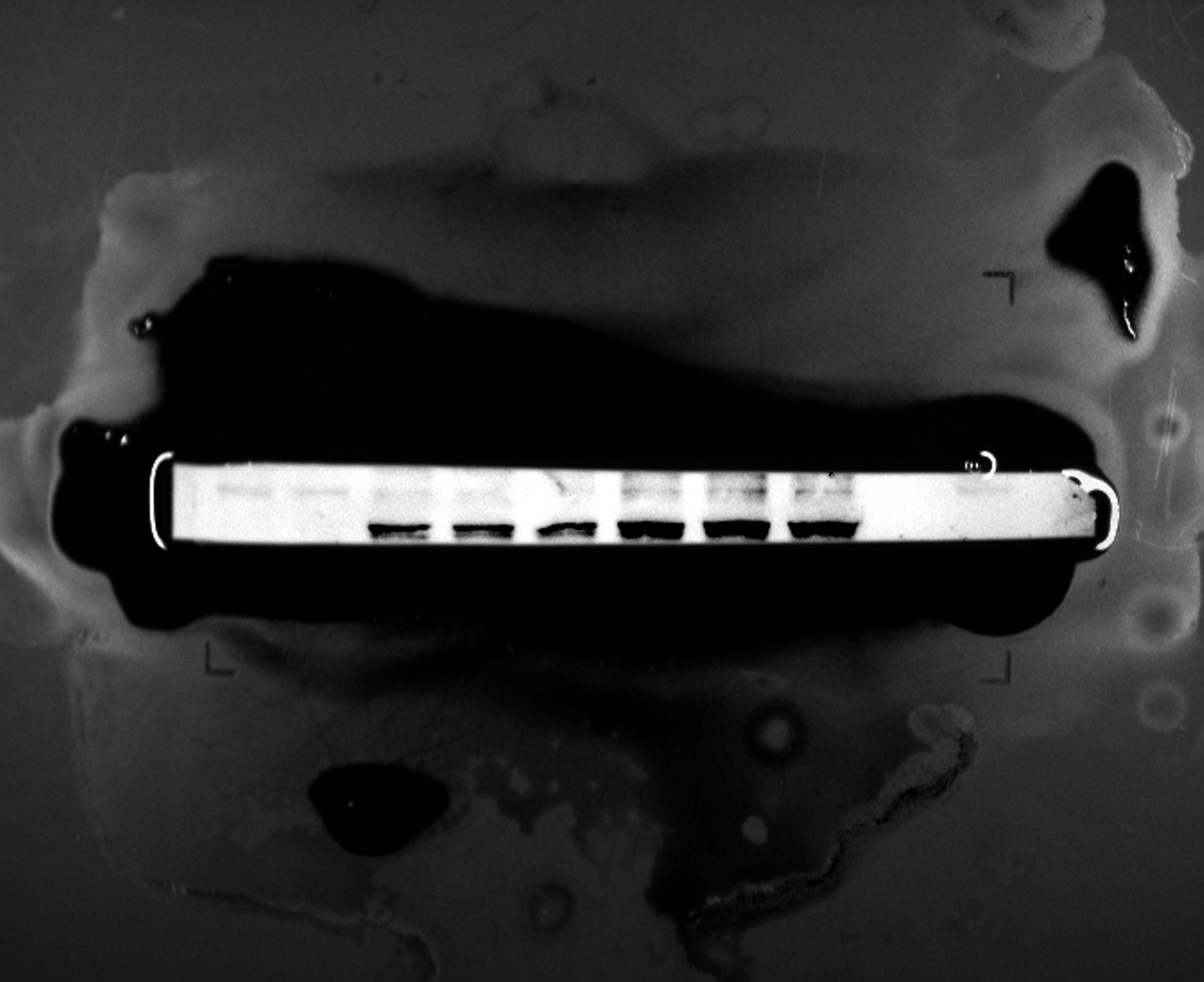

Supplement: Supplementary file 8 [file Data_Sheet_2.ZIP › Scans of the original gels/JUN.Tif]

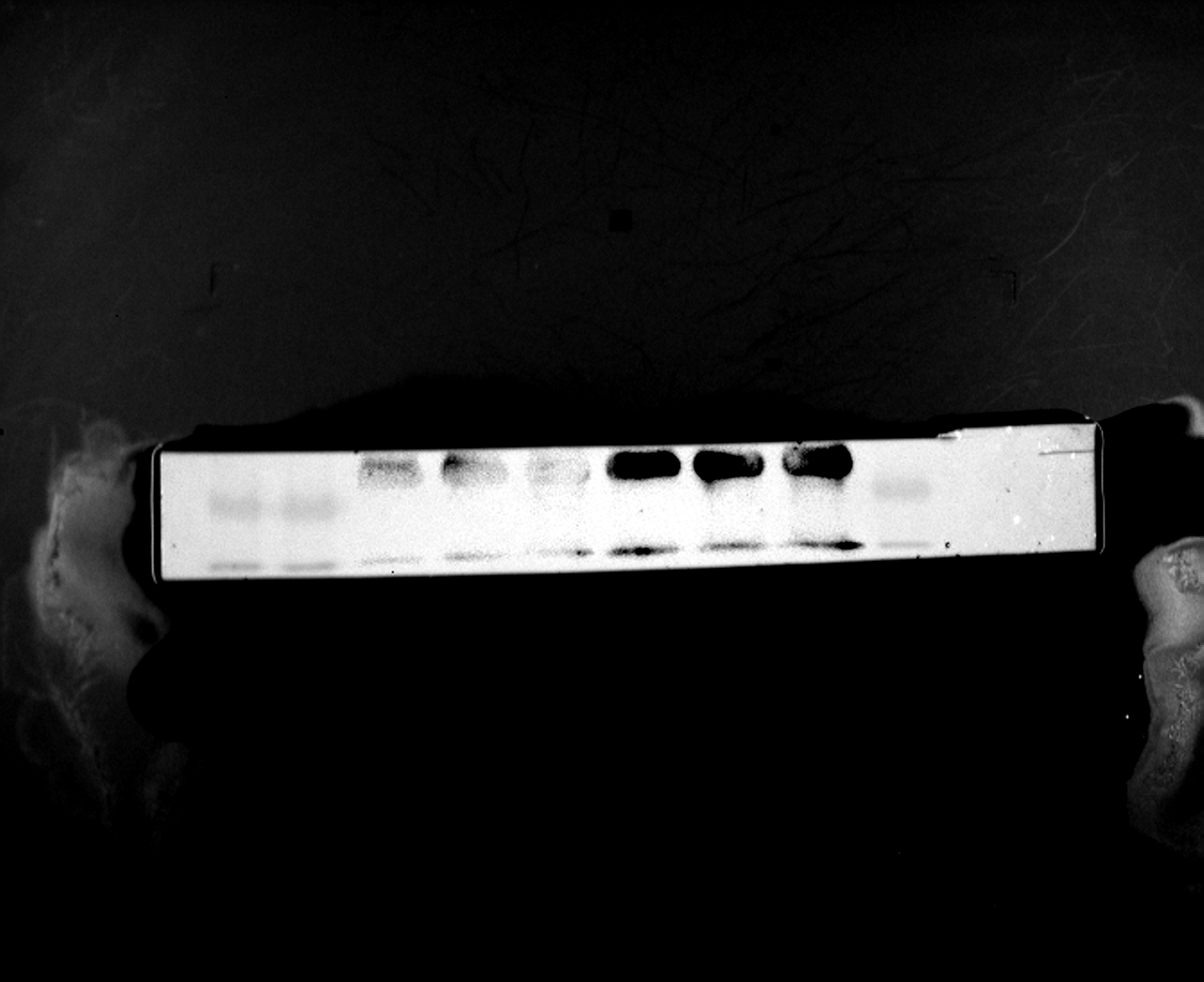

Supplement: Supplementary file 8 [file Data_Sheet_2.ZIP › Scans of the original gels/Timp1.Tif]

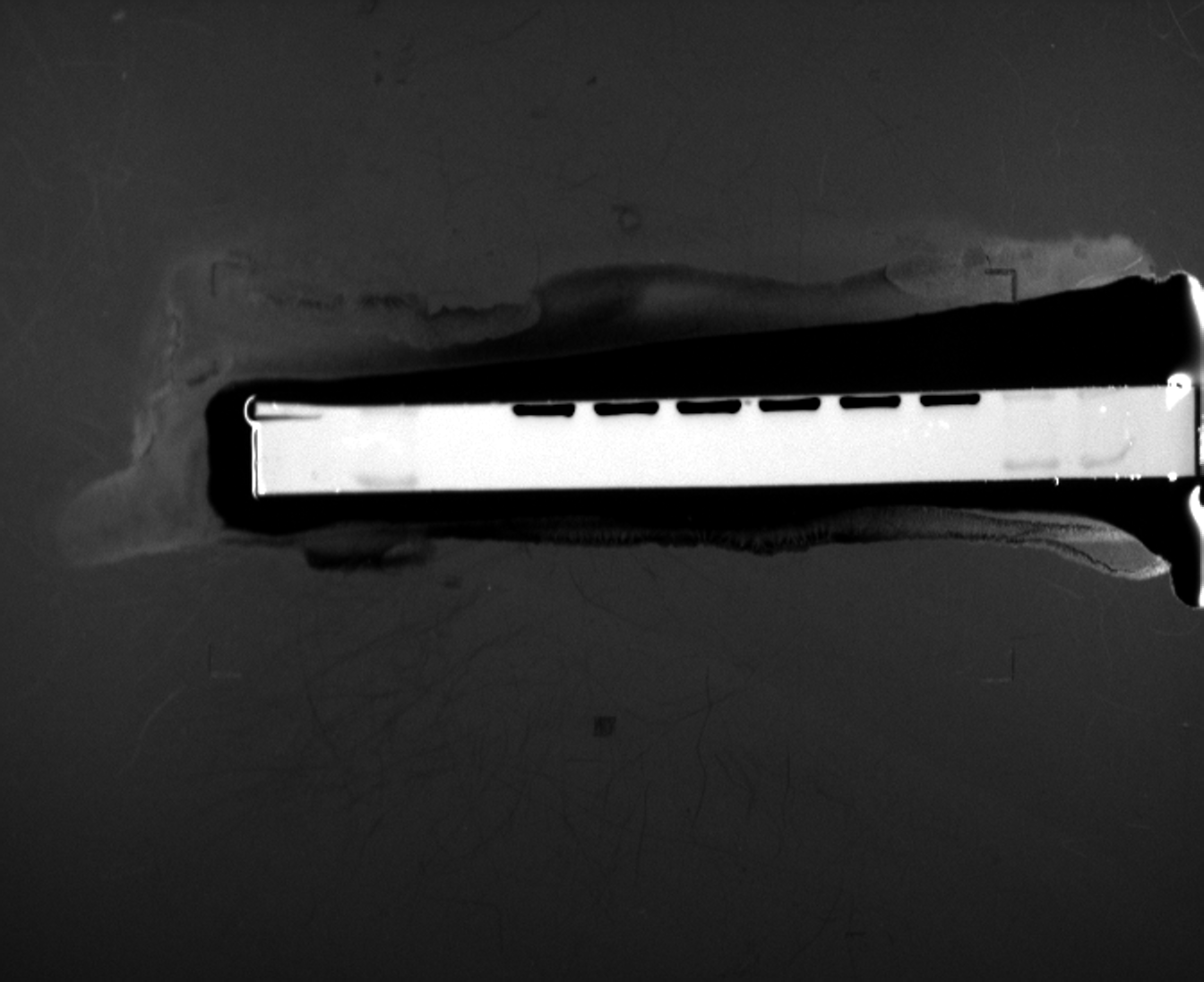

Supplement: Supplementary file 8 [file Data_Sheet_2.ZIP › Scans of the original gels/β-actin.Tif]
