# Supplementary material for: Systematic identification of potential key microRNAs and circRNAs in the dorsal root ganglia of mice with sciatic nerve injury
Source: Front Mol Neurosci. 2023 Mar 14;16:1119164. doi: 10.3389/fnmol.2023.1119164 (PMC10043392; doi:10.3389/fnmol.2023.1119164)
Supplement: Supplementary file 9 [file Data_Sheet_3.ZIP › Relevant code and script files/Relevant code and script files.docx]

**Figure1A-C were generated by the software GEO2R, which is a public software of GEO.** (**https://www.ncbi.nlm.nih.gov/geo/geo2r/?acc=GSE96051)**

# Version info: R 3.2.3, Biobase 2.30.0, GEOquery 2.40.0, limma 3.26.8

################################################################

# Data plots for selected GEO samples

library(GEOquery)

library(limma)

library(umap)

# load series and platform data from GEO

gset <- getGEO("GSE96051", GSEMatrix =TRUE, getGPL=FALSE)

if (length(gset) > 1) idx <- grep("GPL7202", attr(gset, "names")) else idx <- 1

gset <- gset[[idx]]

ex <- exprs(gset)

# log2 transform

qx <- as.numeric(quantile(ex, c(0., 0.25, 0.5, 0.75, 0.99, 1.0), na.rm=T))

LogC <- (qx[5] > 100) ||

(qx[6]-qx[1] > 50 && qx[2] > 0)

if (LogC) { ex[which(ex <= 0)] <- NaN

ex <- log2(ex) }

# box-and-whisker plot

par(mar=c(7,4,2,1))

title <- paste ("GSE96051", "/", annotation(gset), sep ="")

boxplot(ex, boxwex=0.7, notch=T, main=title, outline=FALSE, las=2)

# expression value distribution plot

par(mar=c(4,4,2,1))

title <- paste ("GSE96051", "/", annotation(gset), " value distribution", sep ="")

plotDensities(ex, main=title, legend=F)

# mean-variance trend

ex <- na.omit(ex) # eliminate rows with NAs

plotSA(lmFit(ex), main="Mean variance trend, GSE96051")

# UMAP plot (multi-dimensional scaling)

ex <- ex[!duplicated(ex), ] # remove duplicates

ump <- umap(t(ex), n_neighbors = 4, random_state = 123)

plot(ump$layout, main="UMAP plot, nbrs=4", xlab="", ylab="", pch=20, cex=1.5)

library("maptools") # point labels without overlaps

pointLabel(ump$layout, labels = rownames(ump$layout), method="SANN", cex=0.6)

**Figure1D and Figure1E were drawn by Xiantao (www.xiantao.love). And the data tables are shown in Data of figure1D and Data of figure1E.**

# library(tidyverse)

library(ggplot2)

data <- dat

data <- read.table("~/file.txt", header = T)

head(data)

# gene_name logFC pvalue padj

# 1 TSPAN6 -0.1847277 0.70495937 0.8875868

# 2 TNMD -0.5731570 0.66768318 0.8696220

# 3 DPM1 0.3804519 0.33123387 0.6587634

# 4 SCYL3 -0.6330844 0.22489833 0.5537215

# 5 C1orf112 0.0152763 0.98369157 0.9943630

# 6 FGR -1.0904422 0.01899727 0.1463301

data$col <- "no significant"

data$col[data$padj < 0.05 & data$logFC > 2] <- "Up"

data$col[data$padj < 0.05 & data$logFC < -2] <- "Down"

data$col <- factor(data$col, levels = c("Down", "no significant","Up"))

data$size <- 1

data$size[data$padj < 0.05 & data$logFC > 2] <- 2

data$size[data$padj < 0.05 & data$logFC < -2] <- 2

ggplot() +

geom_point(data = data, aes(logFC, -log10(padj), colour = col, fill = col),

size = data$size) +

scale_colour_manual(values = c("#4DBBD5", "grey", "#E64B35")) +

geom_vline(xintercept = c(-2, 2), color="grey40", linetype=2) +

geom_hline(yintercept = -log10(0.05), color="grey40", linetype=2)

library(tidyverse)

library(ComplexHeatmap)

pheno <- read.table("~/pheno.txt", header = T)

head(pheno)

# sample group other

# 1 sample1 group2 other3

# 2 sample2 group2 other3

# 3 sample3 group2 other3

# 4 sample4 group2 other3

# 5 sample5 group2 other1

# 6 sample6 group2 other1

expr <- read.table("~/expr.txt", header = T, row.names = T)

head(expr[, 1:5])

# sample1 sample2 sample3 sample4 sample5

# Gene1 NA 2.2235 6.8363 3.0055 3.5615

# Gene2 -0.6216 1.0857 2.0330 1.1457 2.0632

# Gene3 -0.2429 5.5259 4.3451 5.0517 -4.9717

# Gene4 4.2098 0.6785 1.6226 0.1889 4.7902

# Gene5 -3.6314 1.6453 2.1495 1.3583 -0.0633

# Gene6 1.6555 -0.1864 -0.1317 -0.0404 2.2153

col = c("#4DBBD5", "#E64B35")

names(col) <- c("group1", "group2")

top <- HeatmapAnnotation(group = pheno$group, col = list(group = col))

Heatmap(as.matrix(t(scale(t(expr)))),

top_annotation = top, show_column_names = F)

**The Figure2 drawing was performed by an online platform for data analysis and visualization (www.bioinformatics.com.cn). The data sheets for Figure2 are detailed in Supplementary Table 2.**

**The Figure3A and Figure3C were performed by the STRING database(https://cn.string-db.org/), and the data table is Supplementary Table 1.**

**The Figure3B was drawn by the Cytoscape 3.9.0 software and the data table is string.tsv.**

**The level of protein expression, autophagy and pain threshold were analyzed by graphpad prism 9 with the student’s t-test and relevant data are shown in Data of WB, PCR, Microscopy and PMWT.** **The myelin sheath damage grade was statistically analyzed using a standard χ2 test.**

**The Figure5A-E were performed by** **Xiantao (www.xiantao.love). And the data tables are shown in Supplementary Table 3.**

# veen

library(tidyverse)

library(ggplot2)

## 2 group ##

# a <- dat[,1][!is.na(dat[,1])]

# b <- dat[,2][!is.na(dat[,2])]

# A <- setdiff(a, b)

# B <- setdiff(b, a)

# AB <- intersect(a, b)

# items <- list(A = A, B = B, AB = AB)

## 3 group ##

# a <- dat[,1][!is.na(dat[,1])]

# b <- dat[,2][!is.na(dat[,2])]

# c <- dat[,3][!is.na(dat[,3])]

# A <- setdiff(a, union(b, c))

# B <- setdiff(b, union(a, c))

# C <- setdiff(c, union(a, b))

# AB <- setdiff(intersect(a, b), c)

# AC <- setdiff(intersect(a, c), b)

# BC <- setdiff(intersect(b, c), a)

# ABC <- intersect(intersect(a, b), c)

# items <- list(A = A, B = B, C = C,

# AB = AB, AC = AC, BC = BC,

# ABC = ABC)

## 4 group ##

# A <- setdiff(a, union(union(b, c), d))

# B <- setdiff(b, union(union(a, c), d))

# C <- setdiff(c, union(union(b, a), d))

# D <- setdiff(d, union(union(b, a), c))

# AB <- setdiff(intersect(a, b), union(c, d))

# AC <- setdiff(intersect(a, c), union(b, d))

# AD <- setdiff(intersect(a, d), union(c, b))

# BC <- setdiff(intersect(c, b), union(a, d))

# BD <- setdiff(intersect(d, b), union(c, a))

# CD <- setdiff(intersect(c, d), union(a, b))

# ABC <- setdiff(intersect(intersect(a, b), c), d)

# ABD <- setdiff(intersect(intersect(a, b), d), c)

# ACD <- setdiff(intersect(intersect(a, d), c), b)

# BCD <- setdiff(intersect(intersect(d, b), c), a)

# ABCD <- intersect(intersect(intersect(a, b), c), d)

# items <- list(A = A, B = B, C = C, D = D, AB = AB, AC = AC,

# AD = AD, BC = BC, BD = BD, CD = CD, ABC = ABC, ABD = ABD,

# ACD = ACD, BCD = BCD, ABCD = ABCD)

## 5 group ##

# A <- setdiff(a, Reduce(union, list(b, c, d, e)))

# B <- setdiff(b, Reduce(union, list(a, c, d, e)))

# C <- setdiff(c, Reduce(union, list(a, b, d, e)))

# D <- setdiff(d, Reduce(union, list(a, b, c, e)))

# E <- setdiff(e, Reduce(union, list(a, b, c, d)))

#

# AB <- setdiff(intersect(a, b), Reduce(union, list(c, d, e)))

# AC <- setdiff(intersect(a, c), Reduce(union, list(b, d, e)))

# AD <- setdiff(intersect(a, d), Reduce(union, list(b, c, e)))

# AE <- setdiff(intersect(a, e), Reduce(union, list(b, c, d)))

# BC <- setdiff(intersect(b, c), Reduce(union, list(a, d, e)))

# BD <- setdiff(intersect(b, d), Reduce(union, list(a, c, e)))

# BE <- setdiff(intersect(b, e), Reduce(union, list(a, c, d)))

# CD <- setdiff(intersect(c, d), Reduce(union, list(a, b, e)))

# CE <- setdiff(intersect(c, e), Reduce(union, list(a, b, d)))

# DE <- setdiff(intersect(d, e), Reduce(union, list(a, b, c)))

#

# ABC <- setdiff(Reduce(intersect, list(a, b, c)), union(d, e))

# ABD <- setdiff(Reduce(intersect, list(a, b, d)), union(c, e))

# ABE <- setdiff(Reduce(intersect, list(a, b, e)), union(c, d))

# ACD <- setdiff(Reduce(intersect, list(a, c, d)), union(b, e))

# ACE <- setdiff(Reduce(intersect, list(a, c, e)), union(b, d))

# ADE <- setdiff(Reduce(intersect, list(a, d, e)), union(b, c))

# BCD <- setdiff(Reduce(intersect, list(b, c, d)), union(a, e))

# BCE <- setdiff(Reduce(intersect, list(b, c, e)), union(a, d))

# BDE <- setdiff(Reduce(intersect, list(b, d, e)), union(a, c))

# CDE <- setdiff(Reduce(intersect, list(c, d, e)), union(a, b))

#

# ABCD <- setdiff(Reduce(intersect, list(a, b, c, d)), e)

# ABCE <- setdiff(Reduce(intersect, list(a, b, c, e)), d)

# ABDE <- setdiff(Reduce(intersect, list(a, b, d, e)), c)

# ACDE <- setdiff(Reduce(intersect, list(a, c, d, e)), b)

# BCDE <- setdiff(Reduce(intersect, list(b, c, d, e)), a)

#

# ABCDE <- Reduce(intersect, list(a, b, c, d, e))

#

# items <- list(A = A, B = B, C = C, D = D, E = E,

# AB = AB, AC = AC, AD = AD, AE = AE,

# BC = BC, BD = BD, BE = BE,

# CD = CD, CE = CE, DE = DE,

# ABC = ABC, ABD = ABD, ABE = ABE, ACD = ACD, ACE = ACE, ADE = ADE,

# BCD = BCD, BCE = BCE, BDE = BDE, CDE = CDE,

# ABCD = ABCD, ABCE = ABCE, ABDE = ABDE,

# ACDE = ACDE, BCDE = BCDE,

# ABCDE = ABCDE)

library(VennDiagram)

venn.diagram(

x = items, filename = "veen.png",

imagetype="png" ,

height = 480,

width = 480,

resolution = 300

)

## the version of ggplot2 is not provided

**The Figure5E** **was drawn by the Cytoscape 3.9.0 software and the data table is Supplementary Table 3.**

**The Figure6 drawing was performed by website (www.bioinformatics.com.cn) to make a sankey diagram of the results. The data sheets for Figure6 are detailed in Supplementary Table 4 and Supplementary Table 5.**

**The Figure9A** **was drawn by the Cytoscape 3.9.0 software and the data table is Supplementary Table 6.** **The Figure9B is drawn by the CSCD database (http://gb.whu.edu.cn/CSCD/).**

**The Figure10A** **was drawn by the Cytoscape 3.9.0 software and the data is shown in Data of Figure 10. The Figure10B was generated by https://starbase.sysu.edu.cn/ and the data is shown in Supplementary Table 6.**
